# Supplementary material for: Green synthesis of silver nanoparticles in aloe vera plant extract prepared by a hydrothermal method and their synergistic antibacterial activity
Source: PeerJ. 2016 Oct 19;4:e2589. doi: 10.7717/peerj.2589 (PMC5075710; doi:10.7717/peerj.2589)
Supplement: Supplemental Information 1 [file peerj-04-2589-s001.docx]

| **UV-vis absorption spectra** | |  |  |  |  |  |
| --- | --- | --- | --- | --- | --- | --- |
| Wavelength (nm) | Absorbance (AU) | | | | | |
|  | 100^o^C for 6h | 150^o^C for 6h | 200^o^C for 6h | 100^o^C for 12h | 150^o^C for 12h | 200^o^C for 12h |
| 300 | 0.238 | 0.152 | 0.137 | 0.27 | 0.149 | 0.114 |
| 300.5 | 0.237 | 0.15 | 0.135 | 0.268 | 0.147 | 0.113 |
| 301 | 0.236 | 0.148 | 0.133 | 0.265 | 0.146 | 0.112 |
| 301.5 | 0.235 | 0.146 | 0.131 | 0.262 | 0.144 | 0.111 |
| 302 | 0.233 | 0.144 | 0.128 | 0.26 | 0.142 | 0.11 |
| 302.5 | 0.232 | 0.142 | 0.126 | 0.257 | 0.14 | 0.108 |
| 303 | 0.23 | 0.139 | 0.124 | 0.254 | 0.138 | 0.107 |
| 303.5 | 0.229 | 0.137 | 0.122 | 0.252 | 0.136 | 0.106 |
| 304 | 0.228 | 0.134 | 0.119 | 0.249 | 0.134 | 0.105 |
| 304.5 | 0.226 | 0.132 | 0.117 | 0.246 | 0.131 | 0.103 |
| 305 | 0.224 | 0.129 | 0.114 | 0.243 | 0.129 | 0.102 |
| 305.5 | 0.223 | 0.127 | 0.111 | 0.239 | 0.126 | 0.1 |
| 306 | 0.22 | 0.123 | 0.109 | 0.236 | 0.123 | 0.099 |
| 306.5 | 0.219 | 0.121 | 0.106 | 0.233 | 0.12 | 0.097 |
| 307 | 0.217 | 0.118 | 0.104 | 0.23 | 0.118 | 0.096 |
| 307.5 | 0.215 | 0.115 | 0.101 | 0.227 | 0.115 | 0.094 |
| 308 | 0.214 | 0.112 | 0.098 | 0.224 | 0.112 | 0.093 |
| 308.5 | 0.212 | 0.109 | 0.095 | 0.221 | 0.109 | 0.092 |
| 309 | 0.21 | 0.106 | 0.092 | 0.218 | 0.107 | 0.09 |
| 309.5 | 0.208 | 0.103 | 0.09 | 0.215 | 0.104 | 0.089 |
| 310 | 0.206 | 0.1 | 0.087 | 0.212 | 0.101 | 0.087 |
| 310.5 | 0.204 | 0.097 | 0.084 | 0.208 | 0.098 | 0.086 |
| 311 | 0.203 | 0.094 | 0.081 | 0.205 | 0.095 | 0.084 |
| 311.5 | 0.201 | 0.092 | 0.079 | 0.202 | 0.092 | 0.083 |
| 312 | 0.2 | 0.089 | 0.077 | 0.2 | 0.09 | 0.081 |
| 312.5 | 0.198 | 0.086 | 0.074 | 0.197 | 0.087 | 0.08 |
| 313 | 0.196 | 0.084 | 0.072 | 0.194 | 0.085 | 0.078 |
| 313.5 | 0.194 | 0.081 | 0.07 | 0.191 | 0.082 | 0.077 |
| 314 | 0.193 | 0.079 | 0.067 | 0.189 | 0.08 | 0.075 |
| 314.5 | 0.191 | 0.077 | 0.065 | 0.186 | 0.078 | 0.074 |
| 315 | 0.19 | 0.075 | 0.063 | 0.184 | 0.076 | 0.073 |
| 315.5 | 0.188 | 0.072 | 0.062 | 0.181 | 0.073 | 0.071 |
| 316 | 0.187 | 0.07 | 0.06 | 0.179 | 0.071 | 0.07 |
| 316.5 | 0.185 | 0.069 | 0.058 | 0.177 | 0.07 | 0.069 |
| 317 | 0.184 | 0.067 | 0.057 | 0.175 | 0.068 | 0.068 |
| 317.5 | 0.183 | 0.066 | 0.055 | 0.173 | 0.067 | 0.068 |
| 318 | 0.181 | 0.065 | 0.054 | 0.171 | 0.066 | 0.067 |
| 318.5 | 0.18 | 0.064 | 0.054 | 0.17 | 0.065 | 0.067 |
| 319 | 0.179 | 0.063 | 0.053 | 0.169 | 0.064 | 0.067 |
| 319.5 | 0.178 | 0.063 | 0.052 | 0.167 | 0.064 | 0.067 |
| 320 | 0.177 | 0.062 | 0.052 | 0.166 | 0.063 | 0.067 |
| 320.5 | 0.176 | 0.062 | 0.052 | 0.165 | 0.063 | 0.067 |
| 321 | 0.175 | 0.062 | 0.052 | 0.164 | 0.063 | 0.067 |
| 321.5 | 0.174 | 0.062 | 0.052 | 0.164 | 0.064 | 0.068 |
| 322 | 0.174 | 0.062 | 0.052 | 0.163 | 0.065 | 0.068 |
| 322.5 | 0.173 | 0.063 | 0.053 | 0.163 | 0.066 | 0.069 |
| 323 | 0.172 | 0.063 | 0.053 | 0.163 | 0.066 | 0.069 |
| 323.5 | 0.172 | 0.064 | 0.054 | 0.162 | 0.067 | 0.07 |
| 324 | 0.171 | 0.065 | 0.055 | 0.163 | 0.068 | 0.07 |
| 324.5 | 0.171 | 0.066 | 0.056 | 0.163 | 0.07 | 0.071 |
| 325 | 0.171 | 0.067 | 0.057 | 0.163 | 0.071 | 0.072 |
| 325.5 | 0.171 | 0.068 | 0.058 | 0.163 | 0.072 | 0.072 |
| 326 | 0.17 | 0.07 | 0.059 | 0.164 | 0.074 | 0.073 |
| 326.5 | 0.17 | 0.072 | 0.061 | 0.164 | 0.076 | 0.073 |
| 327 | 0.171 | 0.073 | 0.063 | 0.165 | 0.078 | 0.074 |
| 327.5 | 0.171 | 0.075 | 0.064 | 0.166 | 0.079 | 0.075 |
| 328 | 0.171 | 0.077 | 0.065 | 0.167 | 0.081 | 0.075 |
| 328.5 | 0.171 | 0.078 | 0.067 | 0.167 | 0.083 | 0.076 |
| 329 | 0.171 | 0.08 | 0.069 | 0.168 | 0.085 | 0.077 |
| 329.5 | 0.172 | 0.082 | 0.071 | 0.17 | 0.087 | 0.077 |
| 330 | 0.173 | 0.084 | 0.073 | 0.171 | 0.089 | 0.078 |
| 330.5 | 0.173 | 0.086 | 0.075 | 0.172 | 0.091 | 0.079 |
| 331 | 0.174 | 0.088 | 0.077 | 0.173 | 0.093 | 0.079 |
| 331.5 | 0.175 | 0.09 | 0.079 | 0.174 | 0.095 | 0.08 |
| 332 | 0.176 | 0.093 | 0.082 | 0.176 | 0.097 | 0.081 |
| 332.5 | 0.176 | 0.095 | 0.084 | 0.177 | 0.099 | 0.082 |
| 333 | 0.177 | 0.097 | 0.086 | 0.178 | 0.101 | 0.082 |
| 333.5 | 0.179 | 0.099 | 0.089 | 0.18 | 0.103 | 0.084 |
| 334 | 0.18 | 0.101 | 0.091 | 0.181 | 0.106 | 0.084 |
| 334.5 | 0.181 | 0.104 | 0.093 | 0.183 | 0.108 | 0.085 |
| 335 | 0.182 | 0.106 | 0.096 | 0.185 | 0.11 | 0.086 |
| 335.5 | 0.184 | 0.109 | 0.098 | 0.186 | 0.112 | 0.087 |
| 336 | 0.185 | 0.111 | 0.101 | 0.188 | 0.114 | 0.088 |
| 336.5 | 0.186 | 0.113 | 0.103 | 0.19 | 0.116 | 0.088 |
| 337 | 0.188 | 0.115 | 0.105 | 0.191 | 0.119 | 0.089 |
| 337.5 | 0.189 | 0.118 | 0.108 | 0.193 | 0.121 | 0.09 |
| 338 | 0.191 | 0.12 | 0.111 | 0.195 | 0.123 | 0.091 |
| 338.5 | 0.192 | 0.122 | 0.113 | 0.196 | 0.125 | 0.092 |
| 339 | 0.194 | 0.125 | 0.116 | 0.198 | 0.127 | 0.092 |
| 339.5 | 0.196 | 0.127 | 0.118 | 0.2 | 0.129 | 0.093 |
| 340 | 0.197 | 0.13 | 0.121 | 0.202 | 0.131 | 0.094 |
| 340.5 | 0.199 | 0.133 | 0.124 | 0.203 | 0.133 | 0.095 |
| 341 | 0.2 | 0.135 | 0.126 | 0.204 | 0.135 | 0.095 |
| 341.5 | 0.202 | 0.137 | 0.129 | 0.206 | 0.137 | 0.096 |
| 342 | 0.203 | 0.139 | 0.131 | 0.208 | 0.139 | 0.097 |
| 342.5 | 0.205 | 0.142 | 0.134 | 0.21 | 0.142 | 0.097 |
| 343 | 0.207 | 0.144 | 0.136 | 0.211 | 0.144 | 0.098 |
| 343.5 | 0.208 | 0.146 | 0.139 | 0.213 | 0.146 | 0.099 |
| 344 | 0.209 | 0.148 | 0.142 | 0.215 | 0.148 | 0.1 |
| 344.5 | 0.211 | 0.151 | 0.145 | 0.216 | 0.15 | 0.101 |
| 345 | 0.212 | 0.153 | 0.147 | 0.218 | 0.152 | 0.102 |
| 345.5 | 0.214 | 0.155 | 0.15 | 0.22 | 0.155 | 0.102 |
| 346 | 0.215 | 0.157 | 0.152 | 0.222 | 0.157 | 0.103 |
| 346.5 | 0.217 | 0.159 | 0.155 | 0.223 | 0.158 | 0.104 |
| 347 | 0.218 | 0.162 | 0.157 | 0.225 | 0.161 | 0.105 |
| 347.5 | 0.219 | 0.164 | 0.16 | 0.227 | 0.163 | 0.106 |
| 348 | 0.221 | 0.167 | 0.163 | 0.228 | 0.165 | 0.107 |
| 348.5 | 0.222 | 0.169 | 0.165 | 0.23 | 0.167 | 0.107 |
| 349 | 0.223 | 0.171 | 0.168 | 0.231 | 0.169 | 0.108 |
| 349.5 | 0.224 | 0.173 | 0.171 | 0.233 | 0.171 | 0.109 |
| 350 | 0.225 | 0.176 | 0.173 | 0.235 | 0.173 | 0.11 |
| 350.5 | 0.226 | 0.178 | 0.176 | 0.236 | 0.175 | 0.11 |
| 351 | 0.227 | 0.18 | 0.178 | 0.238 | 0.177 | 0.111 |
| 351.5 | 0.228 | 0.182 | 0.181 | 0.239 | 0.179 | 0.112 |
| 352 | 0.229 | 0.184 | 0.183 | 0.24 | 0.181 | 0.113 |
| 352.5 | 0.23 | 0.186 | 0.186 | 0.242 | 0.183 | 0.114 |
| 353 | 0.231 | 0.188 | 0.188 | 0.243 | 0.184 | 0.114 |
| 353.5 | 0.231 | 0.19 | 0.19 | 0.244 | 0.186 | 0.115 |
| 354 | 0.232 | 0.192 | 0.192 | 0.246 | 0.188 | 0.115 |
| 354.5 | 0.233 | 0.193 | 0.194 | 0.247 | 0.189 | 0.116 |
| 355 | 0.233 | 0.195 | 0.197 | 0.248 | 0.191 | 0.117 |
| 355.5 | 0.235 | 0.197 | 0.199 | 0.249 | 0.192 | 0.118 |
| 356 | 0.235 | 0.199 | 0.201 | 0.251 | 0.194 | 0.118 |
| 356.5 | 0.236 | 0.201 | 0.203 | 0.252 | 0.195 | 0.119 |
| 357 | 0.237 | 0.202 | 0.205 | 0.253 | 0.196 | 0.119 |
| 357.5 | 0.238 | 0.204 | 0.207 | 0.254 | 0.198 | 0.12 |
| 358 | 0.239 | 0.206 | 0.209 | 0.255 | 0.199 | 0.121 |
| 358.5 | 0.239 | 0.207 | 0.211 | 0.257 | 0.2 | 0.121 |
| 359 | 0.24 | 0.209 | 0.213 | 0.258 | 0.202 | 0.122 |
| 359.5 | 0.241 | 0.21 | 0.215 | 0.259 | 0.203 | 0.122 |
| 360 | 0.242 | 0.212 | 0.218 | 0.26 | 0.205 | 0.123 |
| 360.5 | 0.243 | 0.214 | 0.22 | 0.261 | 0.206 | 0.124 |
| 361 | 0.244 | 0.216 | 0.222 | 0.262 | 0.208 | 0.125 |
| 361.5 | 0.245 | 0.217 | 0.224 | 0.264 | 0.209 | 0.125 |
| 362 | 0.245 | 0.219 | 0.226 | 0.265 | 0.21 | 0.126 |
| 362.5 | 0.246 | 0.221 | 0.228 | 0.266 | 0.212 | 0.126 |
| 363 | 0.247 | 0.222 | 0.229 | 0.267 | 0.213 | 0.127 |
| 363.5 | 0.248 | 0.224 | 0.231 | 0.268 | 0.215 | 0.127 |
| 364 | 0.249 | 0.226 | 0.233 | 0.269 | 0.216 | 0.128 |
| 364.5 | 0.25 | 0.227 | 0.235 | 0.27 | 0.217 | 0.128 |
| 365 | 0.251 | 0.229 | 0.237 | 0.271 | 0.219 | 0.129 |
| 365.5 | 0.252 | 0.231 | 0.239 | 0.273 | 0.221 | 0.13 |
| 366 | 0.253 | 0.233 | 0.241 | 0.274 | 0.222 | 0.13 |
| 366.5 | 0.254 | 0.235 | 0.243 | 0.275 | 0.223 | 0.131 |
| 367 | 0.255 | 0.237 | 0.245 | 0.276 | 0.225 | 0.132 |
| 367.5 | 0.255 | 0.239 | 0.247 | 0.278 | 0.227 | 0.132 |
| 368 | 0.256 | 0.241 | 0.249 | 0.279 | 0.228 | 0.133 |
| 368.5 | 0.257 | 0.242 | 0.251 | 0.28 | 0.23 | 0.134 |
| 369 | 0.258 | 0.244 | 0.253 | 0.281 | 0.231 | 0.134 |
| 369.5 | 0.259 | 0.246 | 0.255 | 0.282 | 0.233 | 0.135 |
| 370 | 0.26 | 0.248 | 0.257 | 0.283 | 0.235 | 0.136 |
| 370.5 | 0.261 | 0.249 | 0.259 | 0.284 | 0.236 | 0.136 |
| 371 | 0.262 | 0.251 | 0.261 | 0.285 | 0.238 | 0.137 |
| 371.5 | 0.263 | 0.253 | 0.263 | 0.286 | 0.239 | 0.138 |
| 372 | 0.263 | 0.255 | 0.265 | 0.287 | 0.241 | 0.138 |
| 372.5 | 0.265 | 0.257 | 0.267 | 0.288 | 0.242 | 0.139 |
| 373 | 0.266 | 0.259 | 0.269 | 0.289 | 0.244 | 0.14 |
| 373.5 | 0.266 | 0.261 | 0.271 | 0.29 | 0.246 | 0.14 |
| 374 | 0.267 | 0.262 | 0.273 | 0.291 | 0.247 | 0.141 |
| 374.5 | 0.268 | 0.264 | 0.275 | 0.292 | 0.249 | 0.142 |
| 375 | 0.269 | 0.266 | 0.277 | 0.293 | 0.251 | 0.142 |
| 375.5 | 0.27 | 0.268 | 0.279 | 0.295 | 0.252 | 0.143 |
| 376 | 0.271 | 0.27 | 0.281 | 0.296 | 0.254 | 0.144 |
| 376.5 | 0.272 | 0.272 | 0.283 | 0.296 | 0.256 | 0.144 |
| 377 | 0.273 | 0.274 | 0.285 | 0.297 | 0.257 | 0.145 |
| 377.5 | 0.274 | 0.276 | 0.287 | 0.298 | 0.259 | 0.146 |
| 378 | 0.274 | 0.278 | 0.289 | 0.299 | 0.26 | 0.146 |
| 378.5 | 0.275 | 0.279 | 0.29 | 0.3 | 0.262 | 0.147 |
| 379 | 0.276 | 0.281 | 0.293 | 0.301 | 0.264 | 0.148 |
| 379.5 | 0.277 | 0.283 | 0.295 | 0.302 | 0.265 | 0.148 |
| 380 | 0.278 | 0.285 | 0.296 | 0.303 | 0.267 | 0.149 |
| 380.5 | 0.278 | 0.287 | 0.298 | 0.304 | 0.268 | 0.15 |
| 381 | 0.279 | 0.289 | 0.3 | 0.305 | 0.27 | 0.15 |
| 381.5 | 0.28 | 0.291 | 0.302 | 0.306 | 0.271 | 0.151 |
| 382 | 0.281 | 0.292 | 0.304 | 0.307 | 0.273 | 0.152 |
| 382.5 | 0.282 | 0.294 | 0.306 | 0.308 | 0.275 | 0.152 |
| 383 | 0.282 | 0.296 | 0.308 | 0.308 | 0.276 | 0.153 |
| 383.5 | 0.283 | 0.298 | 0.31 | 0.309 | 0.278 | 0.154 |
| 384 | 0.284 | 0.299 | 0.312 | 0.31 | 0.279 | 0.154 |
| 384.5 | 0.285 | 0.301 | 0.314 | 0.311 | 0.281 | 0.155 |
| 385 | 0.285 | 0.303 | 0.316 | 0.312 | 0.282 | 0.156 |
| 385.5 | 0.286 | 0.304 | 0.317 | 0.312 | 0.284 | 0.156 |
| 386 | 0.287 | 0.306 | 0.319 | 0.313 | 0.285 | 0.157 |
| 386.5 | 0.288 | 0.308 | 0.321 | 0.314 | 0.287 | 0.158 |
| 387 | 0.288 | 0.309 | 0.323 | 0.315 | 0.288 | 0.158 |
| 387.5 | 0.289 | 0.311 | 0.325 | 0.315 | 0.29 | 0.159 |
| 388 | 0.29 | 0.312 | 0.327 | 0.316 | 0.291 | 0.16 |
| 388.5 | 0.29 | 0.314 | 0.328 | 0.317 | 0.293 | 0.16 |
| 389 | 0.291 | 0.316 | 0.33 | 0.318 | 0.294 | 0.161 |
| 389.5 | 0.292 | 0.317 | 0.332 | 0.318 | 0.296 | 0.162 |
| 390 | 0.292 | 0.319 | 0.334 | 0.319 | 0.297 | 0.162 |
| 390.5 | 0.293 | 0.32 | 0.335 | 0.32 | 0.299 | 0.163 |
| 391 | 0.294 | 0.322 | 0.337 | 0.321 | 0.3 | 0.164 |
| 391.5 | 0.294 | 0.323 | 0.339 | 0.321 | 0.301 | 0.164 |
| 392 | 0.295 | 0.324 | 0.34 | 0.322 | 0.303 | 0.165 |
| 392.5 | 0.296 | 0.326 | 0.342 | 0.322 | 0.304 | 0.166 |
| 393 | 0.296 | 0.327 | 0.343 | 0.323 | 0.305 | 0.166 |
| 393.5 | 0.297 | 0.328 | 0.345 | 0.324 | 0.307 | 0.167 |
| 394 | 0.297 | 0.33 | 0.347 | 0.324 | 0.308 | 0.167 |
| 394.5 | 0.298 | 0.331 | 0.348 | 0.325 | 0.309 | 0.168 |
| 395 | 0.299 | 0.332 | 0.35 | 0.325 | 0.311 | 0.169 |
| 395.5 | 0.299 | 0.333 | 0.351 | 0.326 | 0.312 | 0.169 |
| 396 | 0.3 | 0.334 | 0.353 | 0.327 | 0.313 | 0.17 |
| 396.5 | 0.3 | 0.336 | 0.354 | 0.327 | 0.314 | 0.17 |
| 397 | 0.301 | 0.337 | 0.355 | 0.328 | 0.315 | 0.171 |
| 397.5 | 0.301 | 0.338 | 0.356 | 0.328 | 0.316 | 0.171 |
| 398 | 0.302 | 0.339 | 0.358 | 0.329 | 0.317 | 0.172 |
| 398.5 | 0.302 | 0.34 | 0.359 | 0.329 | 0.319 | 0.172 |
| 399 | 0.303 | 0.341 | 0.36 | 0.33 | 0.319 | 0.173 |
| 399.5 | 0.303 | 0.342 | 0.361 | 0.33 | 0.32 | 0.173 |
| 400 | 0.304 | 0.343 | 0.363 | 0.331 | 0.321 | 0.174 |
| 400.5 | 0.304 | 0.344 | 0.364 | 0.331 | 0.323 | 0.174 |
| 401 | 0.305 | 0.345 | 0.365 | 0.332 | 0.324 | 0.175 |
| 401.5 | 0.305 | 0.345 | 0.366 | 0.332 | 0.324 | 0.175 |
| 402 | 0.306 | 0.346 | 0.367 | 0.332 | 0.325 | 0.176 |
| 402.5 | 0.306 | 0.347 | 0.368 | 0.333 | 0.326 | 0.176 |
| 403 | 0.307 | 0.348 | 0.369 | 0.333 | 0.327 | 0.177 |
| 403.5 | 0.307 | 0.349 | 0.37 | 0.333 | 0.328 | 0.177 |
| 404 | 0.307 | 0.35 | 0.371 | 0.334 | 0.329 | 0.178 |
| 404.5 | 0.308 | 0.35 | 0.372 | 0.334 | 0.33 | 0.178 |
| 405 | 0.308 | 0.351 | 0.373 | 0.335 | 0.331 | 0.179 |
| 405.5 | 0.309 | 0.352 | 0.374 | 0.335 | 0.332 | 0.179 |
| 406 | 0.309 | 0.352 | 0.375 | 0.335 | 0.332 | 0.179 |
| 406.5 | 0.31 | 0.353 | 0.375 | 0.335 | 0.333 | 0.18 |
| 407 | 0.31 | 0.354 | 0.376 | 0.336 | 0.334 | 0.18 |
| 407.5 | 0.31 | 0.354 | 0.377 | 0.336 | 0.335 | 0.181 |
| 408 | 0.311 | 0.355 | 0.378 | 0.336 | 0.335 | 0.181 |
| 408.5 | 0.311 | 0.356 | 0.378 | 0.337 | 0.336 | 0.181 |
| 409 | 0.312 | 0.356 | 0.379 | 0.337 | 0.337 | 0.182 |
| 409.5 | 0.312 | 0.357 | 0.38 | 0.337 | 0.337 | 0.182 |
| 410 | 0.312 | 0.357 | 0.38 | 0.337 | 0.338 | 0.182 |
| 410.5 | 0.313 | 0.358 | 0.381 | 0.337 | 0.339 | 0.183 |
| 411 | 0.313 | 0.358 | 0.381 | 0.338 | 0.339 | 0.183 |
| 411.5 | 0.313 | 0.359 | 0.382 | 0.338 | 0.34 | 0.183 |
| 412 | 0.314 | 0.359 | 0.382 | 0.338 | 0.34 | 0.184 |
| 412.5 | 0.314 | 0.36 | 0.383 | 0.338 | 0.341 | 0.184 |
| 413 | 0.314 | 0.36 | 0.384 | 0.338 | 0.341 | 0.184 |
| 413.5 | 0.315 | 0.36 | 0.384 | 0.338 | 0.342 | 0.185 |
| 414 | 0.315 | 0.361 | 0.384 | 0.339 | 0.342 | 0.185 |
| 414.5 | 0.315 | 0.361 | 0.385 | 0.339 | 0.343 | 0.185 |
| 415 | 0.315 | 0.361 | 0.385 | 0.339 | 0.343 | 0.185 |
| 415.5 | 0.316 | 0.362 | 0.385 | 0.339 | 0.344 | 0.186 |
| 416 | 0.316 | 0.362 | 0.386 | 0.339 | 0.344 | 0.186 |
| 416.5 | 0.316 | 0.362 | 0.386 | 0.339 | 0.345 | 0.186 |
| 417 | 0.317 | 0.363 | 0.387 | 0.339 | 0.345 | 0.186 |
| 417.5 | 0.317 | 0.363 | 0.387 | 0.339 | 0.345 | 0.186 |
| 418 | 0.317 | 0.363 | 0.387 | 0.339 | 0.346 | 0.187 |
| 418.5 | 0.318 | 0.363 | 0.387 | 0.339 | 0.346 | 0.187 |
| 419 | 0.318 | 0.364 | 0.387 | 0.34 | 0.347 | 0.187 |
| 419.5 | 0.318 | 0.364 | 0.388 | 0.34 | 0.347 | 0.187 |
| 420 | 0.318 | 0.364 | 0.388 | 0.34 | 0.347 | 0.187 |
| 420.5 | 0.319 | 0.364 | 0.388 | 0.34 | 0.347 | 0.187 |
| 421 | 0.319 | 0.364 | 0.388 | 0.34 | 0.348 | 0.188 |
| 421.5 | 0.319 | 0.364 | 0.388 | 0.34 | 0.348 | 0.188 |
| 422 | 0.319 | 0.365 | 0.388 | 0.34 | 0.348 | 0.188 |
| 422.5 | 0.32 | 0.365 | 0.388 | 0.34 | 0.348 | 0.188 |
| 423 | 0.32 | 0.365 | 0.388 | 0.34 | 0.349 | 0.188 |
| 423.5 | 0.32 | 0.365 | 0.388 | 0.34 | 0.349 | 0.188 |
| 424 | 0.32 | 0.365 | 0.388 | 0.34 | 0.349 | 0.188 |
| 424.5 | 0.32 | 0.365 | 0.389 | 0.34 | 0.349 | 0.188 |
| 425 | 0.321 | 0.365 | 0.388 | 0.34 | 0.349 | 0.188 |
| 425.5 | 0.321 | 0.365 | 0.388 | 0.34 | 0.349 | 0.188 |
| 426 | 0.321 | 0.365 | 0.388 | 0.34 | 0.349 | 0.188 |
| 426.5 | 0.321 | 0.365 | 0.388 | 0.34 | 0.35 | 0.188 |
| 427 | 0.321 | 0.365 | 0.388 | 0.34 | 0.35 | 0.189 |
| 427.5 | 0.322 | 0.365 | 0.388 | 0.34 | 0.35 | 0.189 |
| 428 | 0.322 | 0.365 | 0.388 | 0.34 | 0.35 | 0.189 |
| 428.5 | 0.322 | 0.365 | 0.388 | 0.34 | 0.35 | 0.189 |
| 429 | 0.322 | 0.365 | 0.388 | 0.34 | 0.35 | 0.189 |
| 429.5 | 0.323 | 0.365 | 0.388 | 0.34 | 0.35 | 0.189 |
| 430 | 0.323 | 0.365 | 0.388 | 0.34 | 0.35 | 0.189 |
| 430.5 | 0.323 | 0.365 | 0.387 | 0.34 | 0.35 | 0.189 |
| 431 | 0.323 | 0.365 | 0.387 | 0.34 | 0.35 | 0.189 |
| 431.5 | 0.323 | 0.365 | 0.387 | 0.34 | 0.35 | 0.189 |
| 432 | 0.323 | 0.365 | 0.387 | 0.34 | 0.35 | 0.189 |
| 432.5 | 0.324 | 0.365 | 0.387 | 0.34 | 0.35 | 0.189 |
| 433 | 0.324 | 0.364 | 0.386 | 0.34 | 0.35 | 0.188 |
| 433.5 | 0.324 | 0.364 | 0.386 | 0.34 | 0.35 | 0.188 |
| 434 | 0.324 | 0.364 | 0.386 | 0.339 | 0.35 | 0.188 |
| 434.5 | 0.324 | 0.364 | 0.386 | 0.339 | 0.35 | 0.188 |
| 435 | 0.324 | 0.364 | 0.385 | 0.339 | 0.35 | 0.188 |
| 435.5 | 0.324 | 0.364 | 0.385 | 0.339 | 0.35 | 0.188 |
| 436 | 0.325 | 0.364 | 0.385 | 0.339 | 0.35 | 0.188 |
| 436.5 | 0.325 | 0.364 | 0.384 | 0.339 | 0.35 | 0.188 |
| 437 | 0.325 | 0.363 | 0.384 | 0.339 | 0.349 | 0.188 |
| 437.5 | 0.325 | 0.363 | 0.384 | 0.339 | 0.349 | 0.188 |
| 438 | 0.325 | 0.363 | 0.383 | 0.339 | 0.349 | 0.188 |
| 438.5 | 0.325 | 0.363 | 0.383 | 0.338 | 0.349 | 0.187 |
| 439 | 0.325 | 0.362 | 0.383 | 0.338 | 0.349 | 0.187 |
| 439.5 | 0.325 | 0.362 | 0.382 | 0.338 | 0.349 | 0.187 |
| 440 | 0.326 | 0.362 | 0.382 | 0.338 | 0.348 | 0.187 |
| 440.5 | 0.326 | 0.362 | 0.381 | 0.338 | 0.348 | 0.187 |
| 441 | 0.326 | 0.362 | 0.381 | 0.338 | 0.348 | 0.187 |
| 441.5 | 0.326 | 0.361 | 0.381 | 0.338 | 0.348 | 0.187 |
| 442 | 0.326 | 0.361 | 0.38 | 0.338 | 0.348 | 0.186 |
| 442.5 | 0.326 | 0.361 | 0.38 | 0.338 | 0.347 | 0.186 |
| 443 | 0.326 | 0.361 | 0.379 | 0.338 | 0.347 | 0.186 |
| 443.5 | 0.326 | 0.36 | 0.379 | 0.337 | 0.347 | 0.186 |
| 444 | 0.326 | 0.36 | 0.378 | 0.337 | 0.347 | 0.186 |
| 444.5 | 0.327 | 0.36 | 0.378 | 0.337 | 0.347 | 0.186 |
| 445 | 0.327 | 0.359 | 0.378 | 0.337 | 0.346 | 0.185 |
| 445.5 | 0.327 | 0.359 | 0.377 | 0.337 | 0.346 | 0.185 |
| 446 | 0.327 | 0.359 | 0.377 | 0.337 | 0.346 | 0.185 |
| 446.5 | 0.327 | 0.359 | 0.376 | 0.337 | 0.346 | 0.185 |
| 447 | 0.327 | 0.358 | 0.376 | 0.336 | 0.345 | 0.185 |
| 447.5 | 0.327 | 0.358 | 0.375 | 0.336 | 0.345 | 0.184 |
| 448 | 0.327 | 0.358 | 0.374 | 0.336 | 0.345 | 0.184 |
| 448.5 | 0.327 | 0.357 | 0.374 | 0.336 | 0.345 | 0.184 |
| 449 | 0.327 | 0.357 | 0.373 | 0.336 | 0.344 | 0.184 |
| 449.5 | 0.328 | 0.357 | 0.373 | 0.336 | 0.344 | 0.184 |
| 450 | 0.328 | 0.356 | 0.372 | 0.336 | 0.344 | 0.183 |
| 450.5 | 0.328 | 0.356 | 0.372 | 0.336 | 0.343 | 0.183 |
| 451 | 0.328 | 0.356 | 0.371 | 0.335 | 0.343 | 0.183 |
| 451.5 | 0.328 | 0.355 | 0.371 | 0.335 | 0.343 | 0.183 |
| 452 | 0.328 | 0.355 | 0.37 | 0.335 | 0.342 | 0.182 |
| 452.5 | 0.328 | 0.355 | 0.37 | 0.335 | 0.342 | 0.182 |
| 453 | 0.328 | 0.354 | 0.369 | 0.335 | 0.342 | 0.182 |
| 453.5 | 0.328 | 0.354 | 0.369 | 0.335 | 0.341 | 0.182 |
| 454 | 0.328 | 0.354 | 0.368 | 0.335 | 0.341 | 0.181 |
| 454.5 | 0.328 | 0.353 | 0.367 | 0.335 | 0.341 | 0.181 |
| 455 | 0.328 | 0.353 | 0.367 | 0.334 | 0.341 | 0.181 |
| 455.5 | 0.329 | 0.352 | 0.366 | 0.334 | 0.34 | 0.181 |
| 456 | 0.329 | 0.352 | 0.366 | 0.334 | 0.34 | 0.181 |
| 456.5 | 0.329 | 0.352 | 0.365 | 0.334 | 0.339 | 0.18 |
| 457 | 0.329 | 0.351 | 0.364 | 0.334 | 0.339 | 0.18 |
| 457.5 | 0.329 | 0.351 | 0.364 | 0.334 | 0.339 | 0.18 |
| 458 | 0.329 | 0.351 | 0.363 | 0.334 | 0.338 | 0.18 |
| 458.5 | 0.329 | 0.35 | 0.363 | 0.333 | 0.338 | 0.179 |
| 459 | 0.329 | 0.35 | 0.362 | 0.333 | 0.338 | 0.179 |
| 459.5 | 0.329 | 0.349 | 0.361 | 0.333 | 0.337 | 0.179 |
| 460 | 0.329 | 0.349 | 0.361 | 0.333 | 0.337 | 0.179 |
| 460.5 | 0.329 | 0.349 | 0.36 | 0.333 | 0.336 | 0.178 |
| 461 | 0.329 | 0.348 | 0.359 | 0.333 | 0.336 | 0.178 |
| 461.5 | 0.329 | 0.348 | 0.359 | 0.333 | 0.336 | 0.178 |
| 462 | 0.329 | 0.347 | 0.358 | 0.332 | 0.335 | 0.177 |
| 462.5 | 0.329 | 0.347 | 0.357 | 0.332 | 0.335 | 0.177 |
| 463 | 0.329 | 0.347 | 0.357 | 0.332 | 0.334 | 0.177 |
| 463.5 | 0.329 | 0.346 | 0.356 | 0.332 | 0.334 | 0.177 |
| 464 | 0.33 | 0.346 | 0.355 | 0.332 | 0.334 | 0.176 |
| 464.5 | 0.33 | 0.345 | 0.355 | 0.332 | 0.333 | 0.176 |
| 465 | 0.33 | 0.345 | 0.354 | 0.332 | 0.333 | 0.176 |
| 465.5 | 0.33 | 0.344 | 0.353 | 0.331 | 0.332 | 0.176 |
| 466 | 0.33 | 0.344 | 0.353 | 0.331 | 0.332 | 0.175 |
| 466.5 | 0.33 | 0.344 | 0.352 | 0.331 | 0.332 | 0.175 |
| 467 | 0.33 | 0.343 | 0.351 | 0.331 | 0.331 | 0.175 |
| 467.5 | 0.33 | 0.343 | 0.351 | 0.331 | 0.331 | 0.174 |
| 468 | 0.33 | 0.342 | 0.35 | 0.331 | 0.33 | 0.174 |
| 468.5 | 0.33 | 0.342 | 0.349 | 0.331 | 0.33 | 0.174 |
| 469 | 0.33 | 0.342 | 0.349 | 0.33 | 0.33 | 0.174 |
| 469.5 | 0.33 | 0.341 | 0.348 | 0.33 | 0.329 | 0.173 |
| 470 | 0.33 | 0.341 | 0.347 | 0.33 | 0.329 | 0.173 |
| 470.5 | 0.33 | 0.34 | 0.347 | 0.33 | 0.328 | 0.173 |
| 471 | 0.33 | 0.34 | 0.346 | 0.33 | 0.328 | 0.173 |
| 471.5 | 0.33 | 0.339 | 0.345 | 0.33 | 0.327 | 0.172 |
| 472 | 0.33 | 0.339 | 0.345 | 0.33 | 0.327 | 0.172 |
| 472.5 | 0.33 | 0.338 | 0.344 | 0.329 | 0.326 | 0.172 |
| 473 | 0.33 | 0.338 | 0.343 | 0.329 | 0.326 | 0.171 |
| 473.5 | 0.33 | 0.338 | 0.342 | 0.329 | 0.326 | 0.171 |
| 474 | 0.33 | 0.337 | 0.342 | 0.329 | 0.325 | 0.171 |
| 474.5 | 0.33 | 0.337 | 0.341 | 0.329 | 0.325 | 0.171 |
| 475 | 0.33 | 0.336 | 0.34 | 0.329 | 0.324 | 0.17 |
| 475.5 | 0.331 | 0.336 | 0.339 | 0.329 | 0.324 | 0.17 |
| 476 | 0.331 | 0.335 | 0.339 | 0.328 | 0.323 | 0.17 |
| 476.5 | 0.331 | 0.335 | 0.338 | 0.328 | 0.323 | 0.169 |
| 477 | 0.331 | 0.335 | 0.337 | 0.328 | 0.322 | 0.169 |
| 477.5 | 0.331 | 0.334 | 0.337 | 0.328 | 0.322 | 0.169 |
| 478 | 0.331 | 0.334 | 0.336 | 0.328 | 0.321 | 0.168 |
| 478.5 | 0.331 | 0.333 | 0.335 | 0.328 | 0.321 | 0.168 |
| 479 | 0.331 | 0.333 | 0.334 | 0.327 | 0.321 | 0.168 |
| 479.5 | 0.331 | 0.332 | 0.334 | 0.327 | 0.32 | 0.168 |
| 480 | 0.331 | 0.332 | 0.333 | 0.327 | 0.32 | 0.167 |
| 480.5 | 0.331 | 0.331 | 0.332 | 0.327 | 0.319 | 0.167 |
| 481 | 0.331 | 0.331 | 0.332 | 0.327 | 0.319 | 0.167 |
| 481.5 | 0.331 | 0.33 | 0.331 | 0.327 | 0.318 | 0.166 |
| 482 | 0.331 | 0.33 | 0.33 | 0.327 | 0.318 | 0.166 |
| 482.5 | 0.331 | 0.33 | 0.329 | 0.327 | 0.317 | 0.166 |
| 483 | 0.331 | 0.329 | 0.328 | 0.326 | 0.317 | 0.165 |
| 483.5 | 0.331 | 0.329 | 0.328 | 0.326 | 0.316 | 0.165 |
| 484 | 0.331 | 0.328 | 0.327 | 0.326 | 0.316 | 0.165 |
| 484.5 | 0.331 | 0.328 | 0.326 | 0.326 | 0.316 | 0.165 |
| 485 | 0.331 | 0.327 | 0.326 | 0.326 | 0.315 | 0.164 |
| 485.5 | 0.331 | 0.327 | 0.325 | 0.326 | 0.315 | 0.164 |
| 486 | 0.331 | 0.326 | 0.324 | 0.326 | 0.314 | 0.164 |
| 486.5 | 0.331 | 0.326 | 0.323 | 0.325 | 0.314 | 0.164 |
| 487 | 0.331 | 0.325 | 0.323 | 0.325 | 0.313 | 0.163 |
| 487.5 | 0.331 | 0.325 | 0.322 | 0.325 | 0.313 | 0.163 |
| 488 | 0.331 | 0.324 | 0.321 | 0.325 | 0.312 | 0.163 |
| 488.5 | 0.331 | 0.324 | 0.32 | 0.325 | 0.312 | 0.163 |
| 489 | 0.331 | 0.324 | 0.32 | 0.325 | 0.312 | 0.162 |
| 489.5 | 0.331 | 0.323 | 0.319 | 0.324 | 0.311 | 0.162 |
| 490 | 0.331 | 0.323 | 0.318 | 0.324 | 0.311 | 0.162 |
| 490.5 | 0.331 | 0.322 | 0.318 | 0.324 | 0.31 | 0.162 |
| 491 | 0.331 | 0.322 | 0.317 | 0.324 | 0.31 | 0.161 |
| 491.5 | 0.331 | 0.321 | 0.316 | 0.324 | 0.309 | 0.161 |
| 492 | 0.331 | 0.321 | 0.315 | 0.324 | 0.309 | 0.161 |
| 492.5 | 0.331 | 0.32 | 0.315 | 0.323 | 0.308 | 0.161 |
| 493 | 0.331 | 0.32 | 0.314 | 0.323 | 0.308 | 0.16 |
| 493.5 | 0.331 | 0.319 | 0.313 | 0.323 | 0.307 | 0.16 |
| 494 | 0.331 | 0.319 | 0.313 | 0.323 | 0.307 | 0.16 |
| 494.5 | 0.331 | 0.318 | 0.312 | 0.323 | 0.307 | 0.159 |
| 495 | 0.331 | 0.318 | 0.311 | 0.323 | 0.306 | 0.159 |
| 495.5 | 0.331 | 0.317 | 0.311 | 0.322 | 0.306 | 0.159 |
| 496 | 0.331 | 0.317 | 0.31 | 0.322 | 0.305 | 0.159 |
| 496.5 | 0.331 | 0.317 | 0.309 | 0.322 | 0.305 | 0.159 |
| 497 | 0.33 | 0.316 | 0.308 | 0.322 | 0.304 | 0.158 |
| 497.5 | 0.33 | 0.316 | 0.307 | 0.322 | 0.304 | 0.158 |
| 498 | 0.33 | 0.315 | 0.307 | 0.322 | 0.304 | 0.158 |
| 498.5 | 0.33 | 0.315 | 0.306 | 0.322 | 0.303 | 0.158 |
| 499 | 0.33 | 0.314 | 0.305 | 0.321 | 0.303 | 0.157 |
| 499.5 | 0.33 | 0.314 | 0.305 | 0.321 | 0.302 | 0.157 |
| 500 | 0.33 | 0.313 | 0.304 | 0.321 | 0.302 | 0.157 |
| 500.5 | 0.33 | 0.313 | 0.303 | 0.321 | 0.301 | 0.156 |
| 501 | 0.33 | 0.312 | 0.303 | 0.321 | 0.301 | 0.156 |
| 501.5 | 0.33 | 0.312 | 0.302 | 0.321 | 0.301 | 0.156 |
| 502 | 0.33 | 0.311 | 0.301 | 0.32 | 0.3 | 0.156 |
| 502.5 | 0.33 | 0.311 | 0.301 | 0.32 | 0.3 | 0.156 |
| 503 | 0.33 | 0.31 | 0.3 | 0.32 | 0.299 | 0.155 |
| 503.5 | 0.33 | 0.31 | 0.299 | 0.32 | 0.299 | 0.155 |
| 504 | 0.33 | 0.31 | 0.299 | 0.32 | 0.298 | 0.155 |
| 504.5 | 0.33 | 0.309 | 0.298 | 0.319 | 0.298 | 0.155 |
| 505 | 0.33 | 0.309 | 0.297 | 0.319 | 0.297 | 0.154 |
| 505.5 | 0.33 | 0.308 | 0.297 | 0.319 | 0.297 | 0.154 |
| 506 | 0.33 | 0.308 | 0.296 | 0.319 | 0.297 | 0.154 |
| 506.5 | 0.33 | 0.307 | 0.295 | 0.319 | 0.296 | 0.154 |
| 507 | 0.33 | 0.307 | 0.295 | 0.319 | 0.296 | 0.153 |
| 507.5 | 0.33 | 0.306 | 0.294 | 0.318 | 0.295 | 0.153 |
| 508 | 0.33 | 0.306 | 0.293 | 0.318 | 0.295 | 0.153 |
| 508.5 | 0.33 | 0.306 | 0.293 | 0.318 | 0.294 | 0.153 |
| 509 | 0.33 | 0.305 | 0.292 | 0.318 | 0.294 | 0.152 |
| 509.5 | 0.329 | 0.305 | 0.291 | 0.318 | 0.293 | 0.152 |
| 510 | 0.329 | 0.304 | 0.29 | 0.317 | 0.293 | 0.152 |
| 510.5 | 0.329 | 0.304 | 0.29 | 0.317 | 0.293 | 0.151 |
| 511 | 0.329 | 0.303 | 0.289 | 0.317 | 0.292 | 0.151 |
| 511.5 | 0.329 | 0.303 | 0.288 | 0.317 | 0.292 | 0.151 |
| 512 | 0.329 | 0.302 | 0.288 | 0.317 | 0.291 | 0.151 |
| 512.5 | 0.329 | 0.302 | 0.287 | 0.316 | 0.291 | 0.15 |
| 513 | 0.329 | 0.301 | 0.286 | 0.316 | 0.291 | 0.15 |
| 513.5 | 0.329 | 0.301 | 0.286 | 0.316 | 0.29 | 0.15 |
| 514 | 0.329 | 0.3 | 0.285 | 0.316 | 0.29 | 0.15 |
| 514.5 | 0.329 | 0.3 | 0.285 | 0.316 | 0.289 | 0.149 |
| 515 | 0.329 | 0.299 | 0.284 | 0.315 | 0.289 | 0.149 |
| 515.5 | 0.328 | 0.299 | 0.283 | 0.315 | 0.288 | 0.149 |
| 516 | 0.328 | 0.299 | 0.283 | 0.315 | 0.288 | 0.149 |
| 516.5 | 0.328 | 0.298 | 0.282 | 0.315 | 0.288 | 0.149 |
| 517 | 0.328 | 0.298 | 0.281 | 0.315 | 0.287 | 0.148 |
| 517.5 | 0.328 | 0.297 | 0.281 | 0.314 | 0.287 | 0.148 |
| 518 | 0.328 | 0.297 | 0.28 | 0.314 | 0.286 | 0.148 |
| 518.5 | 0.328 | 0.296 | 0.279 | 0.314 | 0.286 | 0.148 |
| 519 | 0.328 | 0.296 | 0.279 | 0.314 | 0.286 | 0.147 |
| 519.5 | 0.328 | 0.295 | 0.278 | 0.314 | 0.285 | 0.147 |
| 520 | 0.328 | 0.295 | 0.278 | 0.313 | 0.285 | 0.147 |
| 520.5 | 0.328 | 0.295 | 0.277 | 0.313 | 0.284 | 0.147 |
| 521 | 0.328 | 0.294 | 0.276 | 0.313 | 0.284 | 0.147 |
| 521.5 | 0.328 | 0.294 | 0.276 | 0.313 | 0.284 | 0.146 |
| 522 | 0.328 | 0.293 | 0.275 | 0.313 | 0.283 | 0.146 |
| 522.5 | 0.327 | 0.293 | 0.275 | 0.312 | 0.283 | 0.146 |
| 523 | 0.327 | 0.292 | 0.274 | 0.312 | 0.282 | 0.146 |
| 523.5 | 0.327 | 0.292 | 0.273 | 0.312 | 0.282 | 0.145 |
| 524 | 0.327 | 0.291 | 0.273 | 0.312 | 0.282 | 0.145 |
| 524.5 | 0.327 | 0.291 | 0.272 | 0.312 | 0.281 | 0.145 |
| 525 | 0.327 | 0.29 | 0.272 | 0.311 | 0.281 | 0.145 |
| 525.5 | 0.327 | 0.29 | 0.271 | 0.311 | 0.28 | 0.145 |
| 526 | 0.327 | 0.29 | 0.27 | 0.311 | 0.28 | 0.145 |
| 526.5 | 0.327 | 0.289 | 0.27 | 0.311 | 0.28 | 0.144 |
| 527 | 0.327 | 0.289 | 0.269 | 0.311 | 0.279 | 0.144 |
| 527.5 | 0.327 | 0.288 | 0.269 | 0.31 | 0.279 | 0.144 |
| 528 | 0.327 | 0.288 | 0.268 | 0.31 | 0.278 | 0.144 |
| 528.5 | 0.327 | 0.287 | 0.267 | 0.31 | 0.278 | 0.144 |
| 529 | 0.326 | 0.287 | 0.267 | 0.31 | 0.278 | 0.143 |
| 529.5 | 0.326 | 0.287 | 0.266 | 0.31 | 0.277 | 0.143 |
| 530 | 0.326 | 0.286 | 0.266 | 0.309 | 0.277 | 0.143 |
| 530.5 | 0.326 | 0.286 | 0.265 | 0.309 | 0.276 | 0.143 |
| 531 | 0.326 | 0.285 | 0.265 | 0.309 | 0.276 | 0.143 |
| 531.5 | 0.326 | 0.285 | 0.264 | 0.309 | 0.276 | 0.143 |
| 532 | 0.326 | 0.284 | 0.264 | 0.309 | 0.275 | 0.142 |
| 532.5 | 0.326 | 0.284 | 0.263 | 0.308 | 0.275 | 0.142 |
| 533 | 0.326 | 0.283 | 0.263 | 0.308 | 0.275 | 0.142 |
| 533.5 | 0.326 | 0.283 | 0.262 | 0.308 | 0.274 | 0.142 |
| 534 | 0.326 | 0.283 | 0.262 | 0.308 | 0.274 | 0.142 |
| 534.5 | 0.325 | 0.282 | 0.261 | 0.308 | 0.273 | 0.142 |
| 535 | 0.325 | 0.282 | 0.26 | 0.307 | 0.273 | 0.141 |
| 535.5 | 0.325 | 0.281 | 0.26 | 0.307 | 0.273 | 0.141 |
| 536 | 0.325 | 0.281 | 0.259 | 0.307 | 0.272 | 0.141 |
| 536.5 | 0.325 | 0.28 | 0.259 | 0.307 | 0.272 | 0.141 |
| 537 | 0.325 | 0.28 | 0.258 | 0.307 | 0.272 | 0.141 |
| 537.5 | 0.325 | 0.28 | 0.258 | 0.306 | 0.271 | 0.141 |
| 538 | 0.325 | 0.279 | 0.257 | 0.306 | 0.271 | 0.14 |
| 538.5 | 0.325 | 0.279 | 0.257 | 0.306 | 0.27 | 0.14 |
| 539 | 0.325 | 0.278 | 0.256 | 0.306 | 0.27 | 0.14 |
| 539.5 | 0.325 | 0.278 | 0.256 | 0.305 | 0.27 | 0.14 |
| 540 | 0.324 | 0.278 | 0.255 | 0.305 | 0.269 | 0.14 |
| 540.5 | 0.324 | 0.277 | 0.255 | 0.305 | 0.269 | 0.14 |
| 541 | 0.324 | 0.277 | 0.254 | 0.305 | 0.268 | 0.139 |
| 541.5 | 0.324 | 0.276 | 0.254 | 0.305 | 0.268 | 0.139 |
| 542 | 0.324 | 0.276 | 0.253 | 0.304 | 0.268 | 0.139 |
| 542.5 | 0.324 | 0.276 | 0.253 | 0.304 | 0.267 | 0.139 |
| 543 | 0.324 | 0.275 | 0.252 | 0.304 | 0.267 | 0.139 |
| 543.5 | 0.324 | 0.275 | 0.252 | 0.304 | 0.267 | 0.139 |
| 544 | 0.324 | 0.274 | 0.251 | 0.304 | 0.266 | 0.139 |
| 544.5 | 0.324 | 0.274 | 0.251 | 0.303 | 0.266 | 0.138 |
| 545 | 0.324 | 0.273 | 0.25 | 0.303 | 0.266 | 0.138 |
| 545.5 | 0.323 | 0.273 | 0.25 | 0.303 | 0.265 | 0.138 |
| 546 | 0.323 | 0.273 | 0.249 | 0.303 | 0.265 | 0.138 |
| 546.5 | 0.323 | 0.272 | 0.249 | 0.303 | 0.265 | 0.138 |
| 547 | 0.323 | 0.272 | 0.249 | 0.302 | 0.264 | 0.138 |
| 547.5 | 0.323 | 0.271 | 0.248 | 0.302 | 0.264 | 0.138 |
| 548 | 0.323 | 0.271 | 0.248 | 0.302 | 0.263 | 0.138 |
| 548.5 | 0.323 | 0.271 | 0.247 | 0.302 | 0.263 | 0.137 |
| 549 | 0.323 | 0.27 | 0.247 | 0.301 | 0.263 | 0.137 |
| 549.5 | 0.323 | 0.27 | 0.246 | 0.301 | 0.262 | 0.137 |
| 550 | 0.322 | 0.27 | 0.246 | 0.301 | 0.262 | 0.137 |
| 550.5 | 0.322 | 0.269 | 0.245 | 0.301 | 0.262 | 0.137 |
| 551 | 0.322 | 0.269 | 0.245 | 0.301 | 0.261 | 0.137 |
| 551.5 | 0.322 | 0.268 | 0.244 | 0.3 | 0.261 | 0.137 |
| 552 | 0.322 | 0.268 | 0.244 | 0.3 | 0.261 | 0.136 |
| 552.5 | 0.322 | 0.268 | 0.244 | 0.3 | 0.26 | 0.136 |
| 553 | 0.322 | 0.267 | 0.243 | 0.3 | 0.26 | 0.136 |
| 553.5 | 0.322 | 0.267 | 0.243 | 0.3 | 0.26 | 0.136 |
| 554 | 0.322 | 0.267 | 0.242 | 0.299 | 0.259 | 0.136 |
| 554.5 | 0.322 | 0.266 | 0.242 | 0.299 | 0.259 | 0.136 |
| 555 | 0.321 | 0.266 | 0.242 | 0.299 | 0.259 | 0.136 |
| 555.5 | 0.321 | 0.265 | 0.241 | 0.299 | 0.258 | 0.136 |
| 556 | 0.321 | 0.265 | 0.241 | 0.298 | 0.258 | 0.135 |
| 556.5 | 0.321 | 0.265 | 0.24 | 0.298 | 0.258 | 0.135 |
| 557 | 0.321 | 0.264 | 0.24 | 0.298 | 0.257 | 0.135 |
| 557.5 | 0.321 | 0.264 | 0.239 | 0.298 | 0.257 | 0.135 |
| 558 | 0.321 | 0.264 | 0.239 | 0.297 | 0.257 | 0.135 |
| 558.5 | 0.321 | 0.263 | 0.239 | 0.297 | 0.256 | 0.135 |
| 559 | 0.32 | 0.263 | 0.238 | 0.297 | 0.256 | 0.135 |
| 559.5 | 0.32 | 0.262 | 0.238 | 0.297 | 0.256 | 0.135 |
| 560 | 0.32 | 0.262 | 0.237 | 0.296 | 0.255 | 0.134 |
| 560.5 | 0.32 | 0.262 | 0.237 | 0.296 | 0.255 | 0.134 |
| 561 | 0.32 | 0.261 | 0.237 | 0.296 | 0.255 | 0.134 |
| 561.5 | 0.32 | 0.261 | 0.236 | 0.296 | 0.254 | 0.134 |
| 562 | 0.32 | 0.261 | 0.236 | 0.295 | 0.254 | 0.134 |
| 562.5 | 0.32 | 0.26 | 0.235 | 0.295 | 0.254 | 0.134 |
| 563 | 0.32 | 0.26 | 0.235 | 0.295 | 0.254 | 0.134 |
| 563.5 | 0.319 | 0.26 | 0.235 | 0.295 | 0.253 | 0.134 |
| 564 | 0.319 | 0.259 | 0.234 | 0.294 | 0.253 | 0.134 |
| 564.5 | 0.319 | 0.259 | 0.234 | 0.294 | 0.253 | 0.134 |
| 565 | 0.319 | 0.258 | 0.234 | 0.294 | 0.252 | 0.134 |
| 565.5 | 0.319 | 0.258 | 0.233 | 0.294 | 0.252 | 0.133 |
| 566 | 0.319 | 0.258 | 0.233 | 0.293 | 0.252 | 0.133 |
| 566.5 | 0.318 | 0.257 | 0.232 | 0.293 | 0.251 | 0.133 |
| 567 | 0.318 | 0.257 | 0.232 | 0.293 | 0.251 | 0.133 |
| 567.5 | 0.318 | 0.257 | 0.232 | 0.293 | 0.251 | 0.133 |
| 568 | 0.318 | 0.256 | 0.231 | 0.292 | 0.25 | 0.133 |
| 568.5 | 0.318 | 0.256 | 0.231 | 0.292 | 0.25 | 0.133 |
| 569 | 0.318 | 0.255 | 0.23 | 0.292 | 0.25 | 0.133 |
| 569.5 | 0.318 | 0.255 | 0.23 | 0.292 | 0.249 | 0.133 |
| 570 | 0.317 | 0.255 | 0.23 | 0.291 | 0.249 | 0.132 |
| 570.5 | 0.317 | 0.255 | 0.229 | 0.291 | 0.249 | 0.132 |
| 571 | 0.317 | 0.254 | 0.229 | 0.291 | 0.248 | 0.132 |
| 571.5 | 0.317 | 0.254 | 0.229 | 0.291 | 0.248 | 0.132 |
| 572 | 0.317 | 0.253 | 0.228 | 0.29 | 0.248 | 0.132 |
| 572.5 | 0.317 | 0.253 | 0.228 | 0.29 | 0.248 | 0.132 |
| 573 | 0.317 | 0.253 | 0.228 | 0.29 | 0.247 | 0.132 |
| 573.5 | 0.316 | 0.252 | 0.227 | 0.29 | 0.247 | 0.132 |
| 574 | 0.316 | 0.252 | 0.227 | 0.289 | 0.247 | 0.132 |
| 574.5 | 0.316 | 0.252 | 0.227 | 0.289 | 0.246 | 0.132 |
| 575 | 0.316 | 0.251 | 0.226 | 0.289 | 0.246 | 0.132 |
| 575.5 | 0.316 | 0.251 | 0.226 | 0.288 | 0.246 | 0.132 |
| 576 | 0.316 | 0.251 | 0.226 | 0.288 | 0.245 | 0.131 |
| 576.5 | 0.315 | 0.25 | 0.225 | 0.288 | 0.245 | 0.131 |
| 577 | 0.315 | 0.25 | 0.225 | 0.288 | 0.245 | 0.131 |
| 577.5 | 0.315 | 0.25 | 0.225 | 0.287 | 0.245 | 0.131 |
| 578 | 0.315 | 0.249 | 0.224 | 0.287 | 0.244 | 0.131 |
| 578.5 | 0.315 | 0.249 | 0.224 | 0.287 | 0.244 | 0.131 |
| 579 | 0.315 | 0.249 | 0.223 | 0.287 | 0.244 | 0.131 |
| 579.5 | 0.314 | 0.248 | 0.223 | 0.286 | 0.243 | 0.131 |
| 580 | 0.314 | 0.248 | 0.223 | 0.286 | 0.243 | 0.131 |
| 580.5 | 0.314 | 0.248 | 0.223 | 0.286 | 0.243 | 0.131 |
| 581 | 0.314 | 0.247 | 0.222 | 0.286 | 0.243 | 0.131 |
| 581.5 | 0.314 | 0.247 | 0.222 | 0.285 | 0.242 | 0.131 |
| 582 | 0.313 | 0.247 | 0.222 | 0.285 | 0.242 | 0.131 |
| 582.5 | 0.313 | 0.246 | 0.221 | 0.285 | 0.242 | 0.13 |
| 583 | 0.313 | 0.246 | 0.221 | 0.284 | 0.241 | 0.13 |
| 583.5 | 0.313 | 0.246 | 0.221 | 0.284 | 0.241 | 0.13 |
| 584 | 0.313 | 0.245 | 0.22 | 0.284 | 0.241 | 0.13 |
| 584.5 | 0.313 | 0.245 | 0.22 | 0.284 | 0.24 | 0.13 |
| 585 | 0.312 | 0.245 | 0.22 | 0.283 | 0.24 | 0.13 |
| 585.5 | 0.312 | 0.244 | 0.219 | 0.283 | 0.24 | 0.13 |
| 586 | 0.312 | 0.244 | 0.219 | 0.283 | 0.24 | 0.13 |
| 586.5 | 0.312 | 0.243 | 0.218 | 0.282 | 0.239 | 0.13 |
| 587 | 0.311 | 0.243 | 0.218 | 0.282 | 0.239 | 0.13 |
| 587.5 | 0.311 | 0.243 | 0.218 | 0.282 | 0.239 | 0.13 |
| 588 | 0.311 | 0.243 | 0.218 | 0.282 | 0.238 | 0.13 |
| 588.5 | 0.311 | 0.242 | 0.217 | 0.281 | 0.238 | 0.13 |
| 589 | 0.311 | 0.242 | 0.217 | 0.281 | 0.238 | 0.129 |
| 589.5 | 0.31 | 0.241 | 0.217 | 0.281 | 0.238 | 0.129 |
| 590 | 0.31 | 0.241 | 0.216 | 0.28 | 0.237 | 0.129 |
| 590.5 | 0.31 | 0.241 | 0.216 | 0.28 | 0.237 | 0.129 |
| 591 | 0.31 | 0.24 | 0.216 | 0.28 | 0.237 | 0.129 |
| 591.5 | 0.31 | 0.24 | 0.215 | 0.28 | 0.236 | 0.129 |
| 592 | 0.309 | 0.24 | 0.215 | 0.279 | 0.236 | 0.129 |
| 592.5 | 0.309 | 0.24 | 0.215 | 0.279 | 0.236 | 0.129 |
| 593 | 0.309 | 0.239 | 0.215 | 0.279 | 0.236 | 0.129 |
| 593.5 | 0.309 | 0.239 | 0.214 | 0.278 | 0.235 | 0.129 |
| 594 | 0.309 | 0.239 | 0.214 | 0.278 | 0.235 | 0.129 |
| 594.5 | 0.308 | 0.238 | 0.214 | 0.278 | 0.235 | 0.129 |
| 595 | 0.308 | 0.238 | 0.213 | 0.278 | 0.234 | 0.129 |
| 595.5 | 0.308 | 0.238 | 0.213 | 0.277 | 0.234 | 0.129 |
| 596 | 0.308 | 0.238 | 0.213 | 0.277 | 0.234 | 0.129 |
| 596.5 | 0.308 | 0.237 | 0.213 | 0.277 | 0.234 | 0.129 |
| 597 | 0.307 | 0.237 | 0.212 | 0.277 | 0.233 | 0.129 |
| 597.5 | 0.307 | 0.237 | 0.212 | 0.276 | 0.233 | 0.128 |
| 598 | 0.307 | 0.236 | 0.212 | 0.276 | 0.233 | 0.128 |
| 598.5 | 0.307 | 0.236 | 0.211 | 0.276 | 0.233 | 0.128 |
| 599 | 0.306 | 0.236 | 0.211 | 0.275 | 0.232 | 0.128 |
| 599.5 | 0.306 | 0.235 | 0.211 | 0.275 | 0.232 | 0.128 |
| 600 | 0.306 | 0.235 | 0.211 | 0.275 | 0.232 | 0.128 |
| 600.5 | 0.306 | 0.235 | 0.21 | 0.275 | 0.232 | 0.128 |
| 601 | 0.305 | 0.234 | 0.21 | 0.274 | 0.231 | 0.128 |
| 601.5 | 0.305 | 0.234 | 0.21 | 0.274 | 0.231 | 0.128 |
| 602 | 0.305 | 0.234 | 0.21 | 0.274 | 0.231 | 0.128 |
| 602.5 | 0.305 | 0.233 | 0.209 | 0.274 | 0.231 | 0.128 |
| 603 | 0.305 | 0.233 | 0.209 | 0.273 | 0.23 | 0.128 |
| 603.5 | 0.304 | 0.233 | 0.209 | 0.273 | 0.23 | 0.128 |
| 604 | 0.304 | 0.233 | 0.209 | 0.273 | 0.23 | 0.128 |
| 604.5 | 0.304 | 0.232 | 0.208 | 0.272 | 0.23 | 0.128 |
| 605 | 0.304 | 0.232 | 0.208 | 0.272 | 0.229 | 0.128 |
| 605.5 | 0.303 | 0.232 | 0.208 | 0.272 | 0.229 | 0.128 |
| 606 | 0.303 | 0.231 | 0.207 | 0.272 | 0.229 | 0.128 |
| 606.5 | 0.303 | 0.231 | 0.207 | 0.271 | 0.229 | 0.127 |
| 607 | 0.303 | 0.231 | 0.207 | 0.271 | 0.228 | 0.127 |
| 607.5 | 0.303 | 0.231 | 0.207 | 0.271 | 0.228 | 0.127 |
| 608 | 0.302 | 0.23 | 0.206 | 0.271 | 0.228 | 0.127 |
| 608.5 | 0.302 | 0.23 | 0.206 | 0.27 | 0.227 | 0.127 |
| 609 | 0.302 | 0.23 | 0.206 | 0.27 | 0.227 | 0.127 |
| 609.5 | 0.302 | 0.229 | 0.206 | 0.27 | 0.227 | 0.127 |
| 610 | 0.301 | 0.229 | 0.205 | 0.27 | 0.227 | 0.127 |
| 610.5 | 0.301 | 0.229 | 0.205 | 0.269 | 0.226 | 0.127 |
| 611 | 0.301 | 0.228 | 0.205 | 0.269 | 0.226 | 0.127 |
| 611.5 | 0.301 | 0.228 | 0.205 | 0.269 | 0.226 | 0.127 |
| 612 | 0.3 | 0.228 | 0.204 | 0.268 | 0.226 | 0.127 |
| 612.5 | 0.3 | 0.228 | 0.204 | 0.268 | 0.225 | 0.127 |
| 613 | 0.3 | 0.227 | 0.204 | 0.268 | 0.225 | 0.127 |
| 613.5 | 0.3 | 0.227 | 0.204 | 0.268 | 0.225 | 0.127 |
| 614 | 0.299 | 0.227 | 0.203 | 0.267 | 0.225 | 0.127 |
| 614.5 | 0.299 | 0.227 | 0.203 | 0.267 | 0.225 | 0.127 |
| 615 | 0.299 | 0.226 | 0.203 | 0.267 | 0.224 | 0.127 |
| 615.5 | 0.299 | 0.226 | 0.203 | 0.267 | 0.224 | 0.127 |
| 616 | 0.298 | 0.226 | 0.202 | 0.266 | 0.224 | 0.127 |
| 616.5 | 0.298 | 0.225 | 0.202 | 0.266 | 0.224 | 0.127 |
| 617 | 0.298 | 0.225 | 0.202 | 0.266 | 0.223 | 0.127 |
| 617.5 | 0.298 | 0.225 | 0.202 | 0.266 | 0.223 | 0.127 |
| 618 | 0.297 | 0.225 | 0.201 | 0.265 | 0.223 | 0.127 |
| 618.5 | 0.297 | 0.224 | 0.201 | 0.265 | 0.223 | 0.127 |
| 619 | 0.297 | 0.224 | 0.201 | 0.265 | 0.222 | 0.127 |
| 619.5 | 0.297 | 0.224 | 0.201 | 0.265 | 0.222 | 0.127 |
| 620 | 0.296 | 0.223 | 0.201 | 0.264 | 0.222 | 0.127 |
| 620.5 | 0.296 | 0.223 | 0.2 | 0.264 | 0.222 | 0.127 |
| 621 | 0.296 | 0.223 | 0.2 | 0.264 | 0.221 | 0.127 |
| 621.5 | 0.296 | 0.223 | 0.2 | 0.263 | 0.221 | 0.127 |
| 622 | 0.295 | 0.222 | 0.2 | 0.263 | 0.221 | 0.127 |
| 622.5 | 0.295 | 0.222 | 0.199 | 0.263 | 0.221 | 0.127 |
| 623 | 0.295 | 0.222 | 0.199 | 0.263 | 0.221 | 0.126 |
| 623.5 | 0.295 | 0.222 | 0.199 | 0.262 | 0.22 | 0.126 |
| 624 | 0.295 | 0.221 | 0.199 | 0.262 | 0.22 | 0.126 |
| 624.5 | 0.294 | 0.221 | 0.199 | 0.262 | 0.22 | 0.126 |
| 625 | 0.294 | 0.221 | 0.198 | 0.262 | 0.22 | 0.126 |
| 625.5 | 0.294 | 0.221 | 0.198 | 0.261 | 0.219 | 0.126 |
| 626 | 0.294 | 0.22 | 0.198 | 0.261 | 0.219 | 0.126 |
| 626.5 | 0.293 | 0.22 | 0.198 | 0.261 | 0.219 | 0.126 |
| 627 | 0.293 | 0.22 | 0.197 | 0.261 | 0.219 | 0.126 |
| 627.5 | 0.293 | 0.22 | 0.197 | 0.26 | 0.218 | 0.126 |
| 628 | 0.293 | 0.219 | 0.197 | 0.26 | 0.218 | 0.126 |
| 628.5 | 0.292 | 0.219 | 0.197 | 0.26 | 0.218 | 0.126 |
| 629 | 0.292 | 0.219 | 0.196 | 0.26 | 0.218 | 0.126 |
| 629.5 | 0.292 | 0.219 | 0.196 | 0.26 | 0.218 | 0.126 |
| 630 | 0.292 | 0.218 | 0.196 | 0.259 | 0.217 | 0.126 |
| 630.5 | 0.291 | 0.218 | 0.196 | 0.259 | 0.217 | 0.126 |
| 631 | 0.291 | 0.218 | 0.195 | 0.259 | 0.217 | 0.126 |
| 631.5 | 0.291 | 0.218 | 0.195 | 0.259 | 0.217 | 0.126 |
| 632 | 0.291 | 0.217 | 0.195 | 0.258 | 0.216 | 0.126 |
| 632.5 | 0.29 | 0.217 | 0.195 | 0.258 | 0.216 | 0.126 |
| 633 | 0.29 | 0.217 | 0.195 | 0.258 | 0.216 | 0.126 |
| 633.5 | 0.29 | 0.217 | 0.194 | 0.258 | 0.216 | 0.126 |
| 634 | 0.29 | 0.216 | 0.194 | 0.258 | 0.216 | 0.126 |
| 634.5 | 0.29 | 0.216 | 0.194 | 0.257 | 0.215 | 0.126 |
| 635 | 0.289 | 0.216 | 0.194 | 0.257 | 0.215 | 0.126 |
| 635.5 | 0.289 | 0.216 | 0.193 | 0.257 | 0.215 | 0.126 |
| 636 | 0.289 | 0.215 | 0.193 | 0.257 | 0.215 | 0.126 |
| 636.5 | 0.288 | 0.215 | 0.193 | 0.256 | 0.214 | 0.126 |
| 637 | 0.288 | 0.215 | 0.193 | 0.256 | 0.214 | 0.126 |
| 637.5 | 0.288 | 0.215 | 0.193 | 0.256 | 0.214 | 0.126 |
| 638 | 0.288 | 0.215 | 0.192 | 0.256 | 0.214 | 0.126 |
| 638.5 | 0.288 | 0.214 | 0.192 | 0.255 | 0.214 | 0.126 |
| 639 | 0.287 | 0.214 | 0.192 | 0.255 | 0.214 | 0.126 |
| 639.5 | 0.287 | 0.214 | 0.192 | 0.255 | 0.213 | 0.126 |
| 640 | 0.287 | 0.214 | 0.191 | 0.255 | 0.213 | 0.126 |
| 640.5 | 0.287 | 0.213 | 0.191 | 0.254 | 0.213 | 0.126 |
| 641 | 0.286 | 0.213 | 0.191 | 0.254 | 0.213 | 0.125 |
| 641.5 | 0.286 | 0.213 | 0.191 | 0.254 | 0.212 | 0.126 |
| 642 | 0.286 | 0.213 | 0.191 | 0.254 | 0.212 | 0.126 |
| 642.5 | 0.286 | 0.212 | 0.19 | 0.253 | 0.212 | 0.126 |
| 643 | 0.286 | 0.212 | 0.19 | 0.253 | 0.212 | 0.126 |
| 643.5 | 0.285 | 0.212 | 0.19 | 0.253 | 0.212 | 0.125 |
| 644 | 0.285 | 0.212 | 0.19 | 0.253 | 0.211 | 0.125 |
| 644.5 | 0.285 | 0.212 | 0.19 | 0.252 | 0.211 | 0.125 |
| 645 | 0.285 | 0.211 | 0.189 | 0.252 | 0.211 | 0.125 |
| 645.5 | 0.284 | 0.211 | 0.189 | 0.252 | 0.211 | 0.125 |
| 646 | 0.284 | 0.211 | 0.189 | 0.252 | 0.211 | 0.125 |
| 646.5 | 0.284 | 0.211 | 0.189 | 0.251 | 0.21 | 0.125 |
| 647 | 0.284 | 0.21 | 0.189 | 0.251 | 0.21 | 0.125 |
| 647.5 | 0.283 | 0.21 | 0.188 | 0.251 | 0.21 | 0.125 |
| 648 | 0.283 | 0.21 | 0.188 | 0.251 | 0.21 | 0.125 |
| 648.5 | 0.283 | 0.21 | 0.188 | 0.251 | 0.21 | 0.125 |
| 649 | 0.283 | 0.209 | 0.188 | 0.25 | 0.209 | 0.125 |
| 649.5 | 0.283 | 0.209 | 0.187 | 0.25 | 0.209 | 0.125 |
| 650 | 0.282 | 0.209 | 0.187 | 0.25 | 0.209 | 0.125 |
| 650.5 | 0.282 | 0.209 | 0.187 | 0.25 | 0.209 | 0.125 |
| 651 | 0.282 | 0.209 | 0.187 | 0.249 | 0.209 | 0.125 |
| 651.5 | 0.282 | 0.208 | 0.187 | 0.249 | 0.208 | 0.125 |
| 652 | 0.281 | 0.208 | 0.187 | 0.249 | 0.208 | 0.125 |
| 652.5 | 0.281 | 0.208 | 0.186 | 0.249 | 0.208 | 0.125 |
| 653 | 0.281 | 0.208 | 0.186 | 0.248 | 0.208 | 0.125 |
| 653.5 | 0.281 | 0.208 | 0.186 | 0.248 | 0.208 | 0.125 |
| 654 | 0.28 | 0.207 | 0.186 | 0.248 | 0.208 | 0.125 |
| 654.5 | 0.28 | 0.207 | 0.186 | 0.248 | 0.207 | 0.125 |
| 655 | 0.28 | 0.207 | 0.185 | 0.248 | 0.207 | 0.125 |
| 655.5 | 0.28 | 0.207 | 0.185 | 0.247 | 0.207 | 0.125 |
| 656 | 0.279 | 0.207 | 0.185 | 0.247 | 0.207 | 0.125 |
| 656.5 | 0.279 | 0.206 | 0.185 | 0.247 | 0.206 | 0.125 |
| 657 | 0.279 | 0.206 | 0.185 | 0.247 | 0.206 | 0.125 |
| 657.5 | 0.279 | 0.206 | 0.184 | 0.247 | 0.206 | 0.125 |
| 658 | 0.279 | 0.206 | 0.184 | 0.246 | 0.206 | 0.125 |
| 658.5 | 0.278 | 0.206 | 0.184 | 0.246 | 0.206 | 0.125 |
| 659 | 0.278 | 0.205 | 0.184 | 0.246 | 0.206 | 0.125 |
| 659.5 | 0.278 | 0.205 | 0.184 | 0.246 | 0.205 | 0.125 |
| 660 | 0.278 | 0.205 | 0.184 | 0.245 | 0.205 | 0.125 |
| 660.5 | 0.277 | 0.205 | 0.183 | 0.245 | 0.205 | 0.125 |
| 661 | 0.277 | 0.204 | 0.183 | 0.245 | 0.205 | 0.125 |
| 661.5 | 0.277 | 0.204 | 0.183 | 0.245 | 0.205 | 0.125 |
| 662 | 0.277 | 0.204 | 0.183 | 0.245 | 0.204 | 0.125 |
| 662.5 | 0.276 | 0.204 | 0.183 | 0.245 | 0.204 | 0.125 |
| 663 | 0.276 | 0.204 | 0.182 | 0.244 | 0.204 | 0.125 |
| 663.5 | 0.276 | 0.204 | 0.182 | 0.244 | 0.204 | 0.125 |
| 664 | 0.276 | 0.203 | 0.182 | 0.244 | 0.204 | 0.125 |
| 664.5 | 0.275 | 0.203 | 0.182 | 0.244 | 0.204 | 0.125 |
| 665 | 0.275 | 0.203 | 0.182 | 0.243 | 0.203 | 0.125 |
| 665.5 | 0.275 | 0.203 | 0.182 | 0.243 | 0.203 | 0.125 |
| 666 | 0.275 | 0.202 | 0.181 | 0.243 | 0.203 | 0.125 |
| 666.5 | 0.274 | 0.202 | 0.181 | 0.243 | 0.203 | 0.125 |
| 667 | 0.274 | 0.202 | 0.181 | 0.243 | 0.203 | 0.125 |
| 667.5 | 0.274 | 0.202 | 0.181 | 0.242 | 0.203 | 0.124 |
| 668 | 0.274 | 0.202 | 0.181 | 0.242 | 0.202 | 0.124 |
| 668.5 | 0.273 | 0.201 | 0.18 | 0.242 | 0.202 | 0.125 |
| 669 | 0.273 | 0.201 | 0.18 | 0.242 | 0.202 | 0.124 |
| 669.5 | 0.273 | 0.201 | 0.18 | 0.242 | 0.202 | 0.124 |
| 670 | 0.273 | 0.201 | 0.18 | 0.241 | 0.202 | 0.124 |
| 670.5 | 0.273 | 0.201 | 0.18 | 0.241 | 0.201 | 0.124 |
| 671 | 0.272 | 0.201 | 0.18 | 0.241 | 0.201 | 0.124 |
| 671.5 | 0.272 | 0.2 | 0.179 | 0.241 | 0.201 | 0.124 |
| 672 | 0.272 | 0.2 | 0.179 | 0.24 | 0.201 | 0.124 |
| 672.5 | 0.272 | 0.2 | 0.179 | 0.24 | 0.201 | 0.124 |
| 673 | 0.271 | 0.2 | 0.179 | 0.24 | 0.201 | 0.124 |
| 673.5 | 0.271 | 0.2 | 0.178 | 0.24 | 0.2 | 0.124 |
| 674 | 0.271 | 0.199 | 0.178 | 0.24 | 0.2 | 0.124 |
| 674.5 | 0.271 | 0.199 | 0.178 | 0.239 | 0.2 | 0.124 |
| 675 | 0.271 | 0.199 | 0.178 | 0.239 | 0.2 | 0.124 |
| 675.5 | 0.27 | 0.199 | 0.178 | 0.239 | 0.2 | 0.124 |
| 676 | 0.27 | 0.199 | 0.178 | 0.239 | 0.199 | 0.124 |
| 676.5 | 0.27 | 0.199 | 0.177 | 0.238 | 0.199 | 0.124 |
| 677 | 0.27 | 0.198 | 0.177 | 0.238 | 0.199 | 0.124 |
| 677.5 | 0.27 | 0.198 | 0.177 | 0.238 | 0.199 | 0.124 |
| 678 | 0.269 | 0.198 | 0.177 | 0.238 | 0.199 | 0.124 |
| 678.5 | 0.269 | 0.198 | 0.177 | 0.238 | 0.199 | 0.124 |
| 679 | 0.269 | 0.198 | 0.176 | 0.237 | 0.198 | 0.124 |
| 679.5 | 0.269 | 0.197 | 0.176 | 0.237 | 0.198 | 0.124 |
| 680 | 0.268 | 0.197 | 0.176 | 0.237 | 0.198 | 0.124 |
| 680.5 | 0.268 | 0.197 | 0.176 | 0.237 | 0.198 | 0.124 |
| 681 | 0.268 | 0.197 | 0.176 | 0.237 | 0.198 | 0.124 |
| 681.5 | 0.268 | 0.197 | 0.175 | 0.236 | 0.198 | 0.124 |
| 682 | 0.267 | 0.196 | 0.175 | 0.236 | 0.197 | 0.124 |
| 682.5 | 0.267 | 0.196 | 0.175 | 0.236 | 0.197 | 0.124 |
| 683 | 0.267 | 0.196 | 0.175 | 0.236 | 0.197 | 0.124 |
| 683.5 | 0.267 | 0.196 | 0.175 | 0.236 | 0.197 | 0.124 |
| 684 | 0.266 | 0.196 | 0.174 | 0.235 | 0.197 | 0.124 |
| 684.5 | 0.266 | 0.196 | 0.174 | 0.235 | 0.197 | 0.123 |
| 685 | 0.266 | 0.195 | 0.174 | 0.235 | 0.196 | 0.123 |
| 685.5 | 0.266 | 0.195 | 0.174 | 0.235 | 0.196 | 0.123 |
| 686 | 0.265 | 0.195 | 0.174 | 0.234 | 0.196 | 0.123 |
| 686.5 | 0.265 | 0.195 | 0.173 | 0.234 | 0.196 | 0.123 |
| 687 | 0.265 | 0.195 | 0.173 | 0.234 | 0.196 | 0.123 |
| 687.5 | 0.265 | 0.194 | 0.173 | 0.234 | 0.196 | 0.123 |
| 688 | 0.264 | 0.194 | 0.173 | 0.234 | 0.195 | 0.123 |
| 688.5 | 0.264 | 0.194 | 0.173 | 0.233 | 0.195 | 0.123 |
| 689 | 0.264 | 0.194 | 0.172 | 0.233 | 0.195 | 0.123 |
| 689.5 | 0.264 | 0.194 | 0.172 | 0.233 | 0.195 | 0.123 |
| 690 | 0.264 | 0.194 | 0.172 | 0.233 | 0.195 | 0.123 |
| 690.5 | 0.263 | 0.193 | 0.172 | 0.233 | 0.194 | 0.123 |
| 691 | 0.263 | 0.193 | 0.171 | 0.233 | 0.194 | 0.123 |
| 691.5 | 0.263 | 0.193 | 0.171 | 0.232 | 0.194 | 0.123 |
| 692 | 0.263 | 0.193 | 0.171 | 0.232 | 0.194 | 0.123 |
| 692.5 | 0.262 | 0.193 | 0.171 | 0.232 | 0.194 | 0.123 |
| 693 | 0.262 | 0.192 | 0.171 | 0.232 | 0.194 | 0.123 |
| 693.5 | 0.262 | 0.192 | 0.17 | 0.232 | 0.194 | 0.123 |
| 694 | 0.262 | 0.192 | 0.17 | 0.231 | 0.193 | 0.123 |
| 694.5 | 0.262 | 0.192 | 0.17 | 0.231 | 0.193 | 0.123 |
| 695 | 0.261 | 0.192 | 0.17 | 0.231 | 0.193 | 0.123 |
| 695.5 | 0.261 | 0.192 | 0.17 | 0.231 | 0.193 | 0.123 |
| 696 | 0.261 | 0.191 | 0.169 | 0.231 | 0.193 | 0.122 |
| 696.5 | 0.261 | 0.191 | 0.169 | 0.23 | 0.193 | 0.123 |
| 697 | 0.261 | 0.191 | 0.169 | 0.23 | 0.192 | 0.122 |
| 697.5 | 0.26 | 0.191 | 0.169 | 0.23 | 0.192 | 0.122 |
| 698 | 0.26 | 0.191 | 0.168 | 0.23 | 0.192 | 0.122 |
| 698.5 | 0.26 | 0.191 | 0.168 | 0.23 | 0.192 | 0.122 |
| 699 | 0.26 | 0.19 | 0.168 | 0.229 | 0.192 | 0.122 |
| 699.5 | 0.259 | 0.19 | 0.168 | 0.229 | 0.192 | 0.122 |
| 700 | 0.259 | 0.19 | 0.168 | 0.229 | 0.192 | 0.122 |
| 700.5 | 0.259 | 0.19 | 0.167 | 0.229 | 0.191 | 0.122 |
| 701 | 0.259 | 0.19 | 0.167 | 0.229 | 0.191 | 0.122 |
| 701.5 | 0.259 | 0.19 | 0.167 | 0.229 | 0.191 | 0.122 |
| 702 | 0.258 | 0.189 | 0.167 | 0.228 | 0.191 | 0.122 |
| 702.5 | 0.258 | 0.189 | 0.167 | 0.228 | 0.191 | 0.122 |
| 703 | 0.258 | 0.189 | 0.166 | 0.228 | 0.191 | 0.122 |
| 703.5 | 0.258 | 0.189 | 0.166 | 0.228 | 0.191 | 0.122 |
| 704 | 0.257 | 0.189 | 0.166 | 0.227 | 0.19 | 0.122 |
| 704.5 | 0.257 | 0.189 | 0.166 | 0.227 | 0.19 | 0.122 |
| 705 | 0.257 | 0.188 | 0.165 | 0.227 | 0.19 | 0.122 |
| 705.5 | 0.257 | 0.188 | 0.165 | 0.227 | 0.19 | 0.122 |
| 706 | 0.256 | 0.188 | 0.165 | 0.227 | 0.19 | 0.122 |
| 706.5 | 0.256 | 0.188 | 0.165 | 0.226 | 0.19 | 0.122 |
| 707 | 0.256 | 0.188 | 0.165 | 0.226 | 0.189 | 0.122 |
| 707.5 | 0.256 | 0.188 | 0.164 | 0.226 | 0.189 | 0.122 |
| 708 | 0.256 | 0.187 | 0.164 | 0.226 | 0.189 | 0.122 |
| 708.5 | 0.256 | 0.187 | 0.164 | 0.226 | 0.189 | 0.122 |
| 709 | 0.255 | 0.187 | 0.164 | 0.226 | 0.189 | 0.122 |
| 709.5 | 0.255 | 0.187 | 0.163 | 0.225 | 0.189 | 0.122 |
| 710 | 0.255 | 0.187 | 0.163 | 0.225 | 0.189 | 0.122 |
| 710.5 | 0.255 | 0.187 | 0.163 | 0.225 | 0.188 | 0.122 |
| 711 | 0.254 | 0.186 | 0.163 | 0.225 | 0.188 | 0.122 |
| 711.5 | 0.254 | 0.186 | 0.163 | 0.225 | 0.188 | 0.122 |
| 712 | 0.254 | 0.186 | 0.162 | 0.224 | 0.188 | 0.122 |
| 712.5 | 0.254 | 0.186 | 0.162 | 0.224 | 0.188 | 0.122 |
| 713 | 0.253 | 0.186 | 0.162 | 0.224 | 0.188 | 0.121 |
| 713.5 | 0.253 | 0.186 | 0.162 | 0.224 | 0.187 | 0.121 |
| 714 | 0.253 | 0.185 | 0.162 | 0.224 | 0.187 | 0.121 |
| 714.5 | 0.253 | 0.185 | 0.161 | 0.224 | 0.187 | 0.121 |
| 715 | 0.252 | 0.185 | 0.161 | 0.223 | 0.187 | 0.121 |
| 715.5 | 0.252 | 0.185 | 0.161 | 0.223 | 0.187 | 0.121 |
| 716 | 0.252 | 0.185 | 0.161 | 0.223 | 0.187 | 0.121 |
| 716.5 | 0.252 | 0.185 | 0.161 | 0.223 | 0.186 | 0.121 |
| 717 | 0.252 | 0.184 | 0.16 | 0.223 | 0.186 | 0.121 |
| 717.5 | 0.251 | 0.184 | 0.16 | 0.223 | 0.186 | 0.121 |
| 718 | 0.251 | 0.184 | 0.16 | 0.222 | 0.186 | 0.121 |
| 718.5 | 0.251 | 0.184 | 0.16 | 0.222 | 0.186 | 0.121 |
| 719 | 0.251 | 0.184 | 0.16 | 0.222 | 0.186 | 0.121 |
| 719.5 | 0.251 | 0.184 | 0.159 | 0.222 | 0.186 | 0.121 |
| 720 | 0.25 | 0.183 | 0.159 | 0.222 | 0.185 | 0.121 |
| 720.5 | 0.25 | 0.183 | 0.159 | 0.222 | 0.185 | 0.121 |
| 721 | 0.25 | 0.183 | 0.159 | 0.221 | 0.185 | 0.12 |
| 721.5 | 0.25 | 0.183 | 0.158 | 0.221 | 0.185 | 0.12 |
| 722 | 0.249 | 0.183 | 0.158 | 0.221 | 0.185 | 0.12 |
| 722.5 | 0.249 | 0.183 | 0.158 | 0.221 | 0.185 | 0.12 |
| 723 | 0.249 | 0.182 | 0.158 | 0.221 | 0.185 | 0.12 |
| 723.5 | 0.249 | 0.182 | 0.158 | 0.221 | 0.184 | 0.12 |
| 724 | 0.248 | 0.182 | 0.157 | 0.22 | 0.184 | 0.12 |
| 724.5 | 0.248 | 0.182 | 0.157 | 0.22 | 0.184 | 0.12 |
| 725 | 0.248 | 0.182 | 0.157 | 0.22 | 0.184 | 0.12 |
| 725.5 | 0.248 | 0.182 | 0.157 | 0.22 | 0.184 | 0.12 |
| 726 | 0.247 | 0.181 | 0.157 | 0.22 | 0.184 | 0.12 |
| 726.5 | 0.247 | 0.181 | 0.156 | 0.219 | 0.184 | 0.12 |
| 727 | 0.247 | 0.181 | 0.156 | 0.219 | 0.183 | 0.12 |
| 727.5 | 0.247 | 0.181 | 0.156 | 0.219 | 0.183 | 0.12 |
| 728 | 0.247 | 0.181 | 0.156 | 0.219 | 0.183 | 0.12 |
| 728.5 | 0.247 | 0.181 | 0.156 | 0.218 | 0.183 | 0.12 |
| 729 | 0.247 | 0.18 | 0.155 | 0.218 | 0.183 | 0.12 |
| 729.5 | 0.246 | 0.18 | 0.155 | 0.218 | 0.183 | 0.12 |
| 730 | 0.246 | 0.18 | 0.155 | 0.218 | 0.182 | 0.12 |
| 730.5 | 0.246 | 0.18 | 0.155 | 0.218 | 0.182 | 0.12 |
| 731 | 0.246 | 0.18 | 0.155 | 0.217 | 0.182 | 0.12 |
| 731.5 | 0.245 | 0.179 | 0.154 | 0.217 | 0.182 | 0.119 |
| 732 | 0.245 | 0.179 | 0.154 | 0.217 | 0.182 | 0.119 |
| 732.5 | 0.245 | 0.179 | 0.154 | 0.217 | 0.182 | 0.119 |
| 733 | 0.245 | 0.179 | 0.154 | 0.217 | 0.182 | 0.119 |
| 733.5 | 0.244 | 0.179 | 0.153 | 0.216 | 0.181 | 0.119 |
| 734 | 0.244 | 0.179 | 0.153 | 0.216 | 0.181 | 0.119 |
| 734.5 | 0.244 | 0.178 | 0.153 | 0.216 | 0.181 | 0.119 |
| 735 | 0.244 | 0.178 | 0.153 | 0.216 | 0.181 | 0.119 |
| 735.5 | 0.243 | 0.178 | 0.153 | 0.215 | 0.181 | 0.119 |
| 736 | 0.243 | 0.178 | 0.153 | 0.215 | 0.181 | 0.119 |
| 736.5 | 0.243 | 0.178 | 0.152 | 0.215 | 0.181 | 0.119 |
| 737 | 0.243 | 0.178 | 0.152 | 0.215 | 0.18 | 0.119 |
| 737.5 | 0.242 | 0.178 | 0.152 | 0.215 | 0.18 | 0.119 |
| 738 | 0.242 | 0.177 | 0.152 | 0.215 | 0.18 | 0.119 |
| 738.5 | 0.242 | 0.177 | 0.151 | 0.214 | 0.18 | 0.119 |
| 739 | 0.242 | 0.177 | 0.151 | 0.214 | 0.18 | 0.119 |
| 739.5 | 0.241 | 0.177 | 0.151 | 0.214 | 0.18 | 0.119 |
| 740 | 0.241 | 0.177 | 0.151 | 0.214 | 0.18 | 0.119 |
| 740.5 | 0.241 | 0.177 | 0.151 | 0.214 | 0.18 | 0.119 |
| 741 | 0.241 | 0.176 | 0.15 | 0.214 | 0.179 | 0.119 |
| 741.5 | 0.24 | 0.176 | 0.15 | 0.213 | 0.179 | 0.118 |
| 742 | 0.24 | 0.176 | 0.15 | 0.213 | 0.179 | 0.118 |
| 742.5 | 0.24 | 0.176 | 0.15 | 0.213 | 0.179 | 0.118 |
| 743 | 0.24 | 0.176 | 0.15 | 0.213 | 0.179 | 0.118 |
| 743.5 | 0.24 | 0.176 | 0.15 | 0.213 | 0.179 | 0.118 |
| 744 | 0.239 | 0.175 | 0.149 | 0.213 | 0.179 | 0.118 |
| 744.5 | 0.239 | 0.175 | 0.149 | 0.213 | 0.178 | 0.118 |
| 745 | 0.239 | 0.175 | 0.149 | 0.212 | 0.178 | 0.118 |
| 745.5 | 0.239 | 0.175 | 0.149 | 0.212 | 0.178 | 0.118 |
| 746 | 0.239 | 0.175 | 0.149 | 0.212 | 0.178 | 0.118 |
| 746.5 | 0.238 | 0.175 | 0.148 | 0.212 | 0.178 | 0.118 |
| 747 | 0.238 | 0.175 | 0.148 | 0.212 | 0.178 | 0.118 |
| 747.5 | 0.238 | 0.174 | 0.148 | 0.212 | 0.178 | 0.118 |
| 748 | 0.238 | 0.174 | 0.148 | 0.211 | 0.178 | 0.118 |
| 748.5 | 0.238 | 0.174 | 0.148 | 0.211 | 0.177 | 0.118 |
| 749 | 0.238 | 0.174 | 0.147 | 0.211 | 0.177 | 0.118 |
| 749.5 | 0.237 | 0.174 | 0.147 | 0.211 | 0.177 | 0.118 |
| 750 | 0.237 | 0.174 | 0.147 | 0.211 | 0.177 | 0.118 |
| 750.5 | 0.237 | 0.173 | 0.147 | 0.21 | 0.177 | 0.118 |
| 751 | 0.237 | 0.173 | 0.147 | 0.21 | 0.177 | 0.118 |
| 751.5 | 0.236 | 0.173 | 0.146 | 0.21 | 0.177 | 0.118 |
| 752 | 0.236 | 0.173 | 0.146 | 0.21 | 0.177 | 0.118 |
| 752.5 | 0.236 | 0.173 | 0.146 | 0.21 | 0.176 | 0.118 |
| 753 | 0.236 | 0.173 | 0.146 | 0.209 | 0.176 | 0.117 |
| 753.5 | 0.236 | 0.173 | 0.146 | 0.209 | 0.176 | 0.117 |
| 754 | 0.235 | 0.172 | 0.146 | 0.209 | 0.176 | 0.117 |
| 754.5 | 0.235 | 0.172 | 0.145 | 0.209 | 0.176 | 0.117 |
| 755 | 0.235 | 0.172 | 0.145 | 0.209 | 0.176 | 0.117 |
| 755.5 | 0.235 | 0.172 | 0.145 | 0.208 | 0.176 | 0.117 |
| 756 | 0.235 | 0.172 | 0.145 | 0.208 | 0.176 | 0.117 |
| 756.5 | 0.234 | 0.172 | 0.145 | 0.208 | 0.175 | 0.117 |
| 757 | 0.234 | 0.171 | 0.145 | 0.208 | 0.175 | 0.117 |
| 757.5 | 0.234 | 0.171 | 0.144 | 0.208 | 0.175 | 0.117 |
| 758 | 0.234 | 0.171 | 0.144 | 0.207 | 0.175 | 0.117 |
| 758.5 | 0.233 | 0.171 | 0.144 | 0.207 | 0.175 | 0.117 |
| 759 | 0.233 | 0.171 | 0.144 | 0.207 | 0.175 | 0.117 |
| 759.5 | 0.233 | 0.171 | 0.144 | 0.207 | 0.175 | 0.117 |
| 760 | 0.233 | 0.171 | 0.143 | 0.207 | 0.175 | 0.117 |
| 760.5 | 0.233 | 0.17 | 0.143 | 0.206 | 0.174 | 0.116 |
| 761 | 0.232 | 0.17 | 0.143 | 0.206 | 0.174 | 0.116 |
| 761.5 | 0.232 | 0.17 | 0.143 | 0.206 | 0.174 | 0.116 |
| 762 | 0.232 | 0.17 | 0.143 | 0.206 | 0.174 | 0.116 |
| 762.5 | 0.232 | 0.17 | 0.143 | 0.206 | 0.174 | 0.116 |
| 763 | 0.231 | 0.17 | 0.142 | 0.205 | 0.174 | 0.116 |
| 763.5 | 0.231 | 0.169 | 0.142 | 0.205 | 0.174 | 0.116 |
| 764 | 0.231 | 0.169 | 0.142 | 0.205 | 0.174 | 0.116 |
| 764.5 | 0.231 | 0.169 | 0.142 | 0.205 | 0.173 | 0.116 |
| 765 | 0.231 | 0.169 | 0.142 | 0.205 | 0.173 | 0.116 |
| 765.5 | 0.23 | 0.169 | 0.141 | 0.205 | 0.173 | 0.116 |
| 766 | 0.23 | 0.169 | 0.141 | 0.204 | 0.173 | 0.116 |
| 766.5 | 0.23 | 0.169 | 0.141 | 0.204 | 0.173 | 0.116 |
| 767 | 0.23 | 0.168 | 0.141 | 0.204 | 0.173 | 0.116 |
| 767.5 | 0.23 | 0.168 | 0.141 | 0.204 | 0.173 | 0.116 |
| 768 | 0.229 | 0.168 | 0.141 | 0.204 | 0.173 | 0.115 |
| 768.5 | 0.229 | 0.168 | 0.14 | 0.204 | 0.172 | 0.115 |
| 769 | 0.229 | 0.168 | 0.14 | 0.204 | 0.172 | 0.115 |
| 769.5 | 0.229 | 0.168 | 0.14 | 0.203 | 0.172 | 0.115 |
| 770 | 0.228 | 0.168 | 0.14 | 0.203 | 0.172 | 0.115 |
| 770.5 | 0.228 | 0.168 | 0.14 | 0.203 | 0.172 | 0.115 |
| 771 | 0.228 | 0.167 | 0.14 | 0.203 | 0.172 | 0.115 |
| 771.5 | 0.228 | 0.167 | 0.139 | 0.202 | 0.172 | 0.115 |
| 772 | 0.228 | 0.167 | 0.139 | 0.202 | 0.172 | 0.115 |
| 772.5 | 0.227 | 0.167 | 0.139 | 0.202 | 0.171 | 0.115 |
| 773 | 0.227 | 0.167 | 0.139 | 0.202 | 0.171 | 0.115 |
| 773.5 | 0.227 | 0.167 | 0.139 | 0.202 | 0.171 | 0.115 |
| 774 | 0.227 | 0.167 | 0.138 | 0.201 | 0.171 | 0.115 |
| 774.5 | 0.227 | 0.166 | 0.138 | 0.201 | 0.171 | 0.115 |
| 775 | 0.227 | 0.166 | 0.138 | 0.201 | 0.171 | 0.115 |
| 775.5 | 0.226 | 0.166 | 0.138 | 0.201 | 0.171 | 0.115 |
| 776 | 0.226 | 0.166 | 0.138 | 0.201 | 0.171 | 0.115 |
| 776.5 | 0.226 | 0.166 | 0.137 | 0.201 | 0.17 | 0.115 |
| 777 | 0.226 | 0.166 | 0.137 | 0.2 | 0.17 | 0.115 |
| 777.5 | 0.226 | 0.166 | 0.137 | 0.2 | 0.17 | 0.115 |
| 778 | 0.225 | 0.165 | 0.137 | 0.2 | 0.17 | 0.115 |
| 778.5 | 0.225 | 0.165 | 0.137 | 0.2 | 0.17 | 0.115 |
| 779 | 0.225 | 0.165 | 0.137 | 0.2 | 0.17 | 0.115 |
| 779.5 | 0.225 | 0.165 | 0.136 | 0.2 | 0.17 | 0.115 |
| 780 | 0.225 | 0.165 | 0.136 | 0.199 | 0.17 | 0.115 |
| 780.5 | 0.224 | 0.165 | 0.136 | 0.199 | 0.17 | 0.115 |
| 781 | 0.224 | 0.165 | 0.136 | 0.199 | 0.169 | 0.115 |
| 781.5 | 0.224 | 0.164 | 0.136 | 0.199 | 0.169 | 0.114 |
| 782 | 0.224 | 0.164 | 0.136 | 0.199 | 0.169 | 0.114 |
| 782.5 | 0.223 | 0.164 | 0.135 | 0.199 | 0.169 | 0.114 |
| 783 | 0.223 | 0.164 | 0.135 | 0.198 | 0.169 | 0.114 |
| 783.5 | 0.223 | 0.164 | 0.135 | 0.198 | 0.169 | 0.114 |
| 784 | 0.223 | 0.164 | 0.135 | 0.198 | 0.169 | 0.114 |
| 784.5 | 0.223 | 0.163 | 0.135 | 0.198 | 0.169 | 0.114 |
| 785 | 0.223 | 0.163 | 0.135 | 0.198 | 0.168 | 0.114 |
| 785.5 | 0.222 | 0.163 | 0.134 | 0.198 | 0.168 | 0.114 |
| 786 | 0.222 | 0.163 | 0.134 | 0.197 | 0.168 | 0.114 |
| 786.5 | 0.222 | 0.163 | 0.134 | 0.197 | 0.168 | 0.114 |
| 787 | 0.221 | 0.163 | 0.134 | 0.197 | 0.168 | 0.114 |
| 787.5 | 0.221 | 0.162 | 0.134 | 0.197 | 0.168 | 0.114 |
| 788 | 0.221 | 0.162 | 0.134 | 0.197 | 0.168 | 0.114 |
| 788.5 | 0.221 | 0.162 | 0.133 | 0.197 | 0.168 | 0.114 |
| 789 | 0.221 | 0.162 | 0.133 | 0.196 | 0.167 | 0.114 |
| 789.5 | 0.221 | 0.162 | 0.133 | 0.196 | 0.167 | 0.114 |
| 790 | 0.221 | 0.162 | 0.133 | 0.196 | 0.167 | 0.114 |
| 790.5 | 0.22 | 0.161 | 0.133 | 0.196 | 0.167 | 0.114 |
| 791 | 0.22 | 0.161 | 0.133 | 0.196 | 0.167 | 0.114 |
| 791.5 | 0.22 | 0.161 | 0.132 | 0.196 | 0.167 | 0.114 |
| 792 | 0.22 | 0.161 | 0.132 | 0.195 | 0.167 | 0.114 |
| 792.5 | 0.219 | 0.161 | 0.132 | 0.195 | 0.167 | 0.114 |
| 793 | 0.219 | 0.161 | 0.132 | 0.195 | 0.166 | 0.114 |
| 793.5 | 0.219 | 0.16 | 0.132 | 0.195 | 0.166 | 0.114 |
| 794 | 0.219 | 0.16 | 0.132 | 0.195 | 0.166 | 0.114 |
| 794.5 | 0.218 | 0.16 | 0.131 | 0.195 | 0.166 | 0.114 |
| 795 | 0.218 | 0.16 | 0.131 | 0.194 | 0.166 | 0.114 |
| 795.5 | 0.218 | 0.16 | 0.131 | 0.194 | 0.166 | 0.114 |
| 796 | 0.217 | 0.16 | 0.131 | 0.194 | 0.166 | 0.114 |
| 796.5 | 0.217 | 0.159 | 0.131 | 0.194 | 0.166 | 0.113 |
| 797 | 0.217 | 0.159 | 0.131 | 0.194 | 0.165 | 0.113 |
| 797.5 | 0.217 | 0.159 | 0.13 | 0.193 | 0.165 | 0.113 |
| 798 | 0.217 | 0.159 | 0.13 | 0.193 | 0.165 | 0.113 |
| 798.5 | 0.217 | 0.159 | 0.13 | 0.193 | 0.165 | 0.113 |
| 799 | 0.216 | 0.159 | 0.13 | 0.193 | 0.165 | 0.113 |
| 799.5 | 0.216 | 0.158 | 0.13 | 0.193 | 0.165 | 0.113 |
| 800 | 0.216 | 0.158 | 0.13 | 0.193 | 0.165 | 0.113 |
